# Six new species of *Begonia* from Guangxi, China

**DOI:** 10.1186/s40529-020-00298-y

**Published:** 2020-07-30

**Authors:** Yan Liu, Yu-Hsin Tseng, Hsun-An Yang, Ai-Qun Hu, Wei-Bin Xu, Che-Wei Lin, Yoshiko Kono, Chiung-Chih Chang, Ching-I Peng, Kuo-Fang Chung

**Affiliations:** 1 Guangxi Key Laboratory of Plant Conservation and Restoration Ecology in Karst Terrain, Guangxi Institute of Botany, Guangxi Zhuang Autonomous Region, Chinese Academy of Sciences, Guilin, Guangxi China; 2 Research Museum and Herbarium (HAST), Biodiversity Research Center, Academia Sinica, Taipei, Taiwan; 3 Herbarium (TAIF), Taiwan Forestry Research Institute, Taipei, Taiwan; 4 The Community Center for the Advancement of Education and Research, University of Kochi, Kochi, Japan

**Keywords:** *Begonia* sect. *Coelocentrum*, *Begonia* sect. *Platycentrum*, Phylogenetics, Sino-Vietnamese limestone karsts, Taxonomy

## Abstract

**Background:**

With currently 1980 described species, the mega-diverse *Begonia* is now perhaps the 5th largest flowering plant genus, expanding rapidly from ca. 900 species in 1997 to its current size in merely two decades. In continuation of our studies of Asian *Begonia*, we report six additional new species from Guangxi, the region/province harboring the second richest *Begonia* flora of China.

**Results:**

Based on morphological and molecular data, the new species *B. aurora* belongs to *Begonia* sect. *Platycentrum*, while the other five new species (viz. *B. larvata*, *B. longiornithophylla*, *B. lui*, *B. scabrifolia*, and *B. zhuoyuniae*) are members of Sect. *Coelocentrum*. Somatic chromosome numbers of *B. longiornithophylla* and *B. zhuoyuniae* at metaphase were counted as 2*n* = 30, consistent with previously reports for Sect. *Coelocentrum*.

**Conclusions:**

With the addition of the six new species, the total number of *Begonia* species in Guangxi increases from 86 to 92. Detailed description, line drawings, and color plates are provided to aid in identification.

## Background

**Fig. 1:**
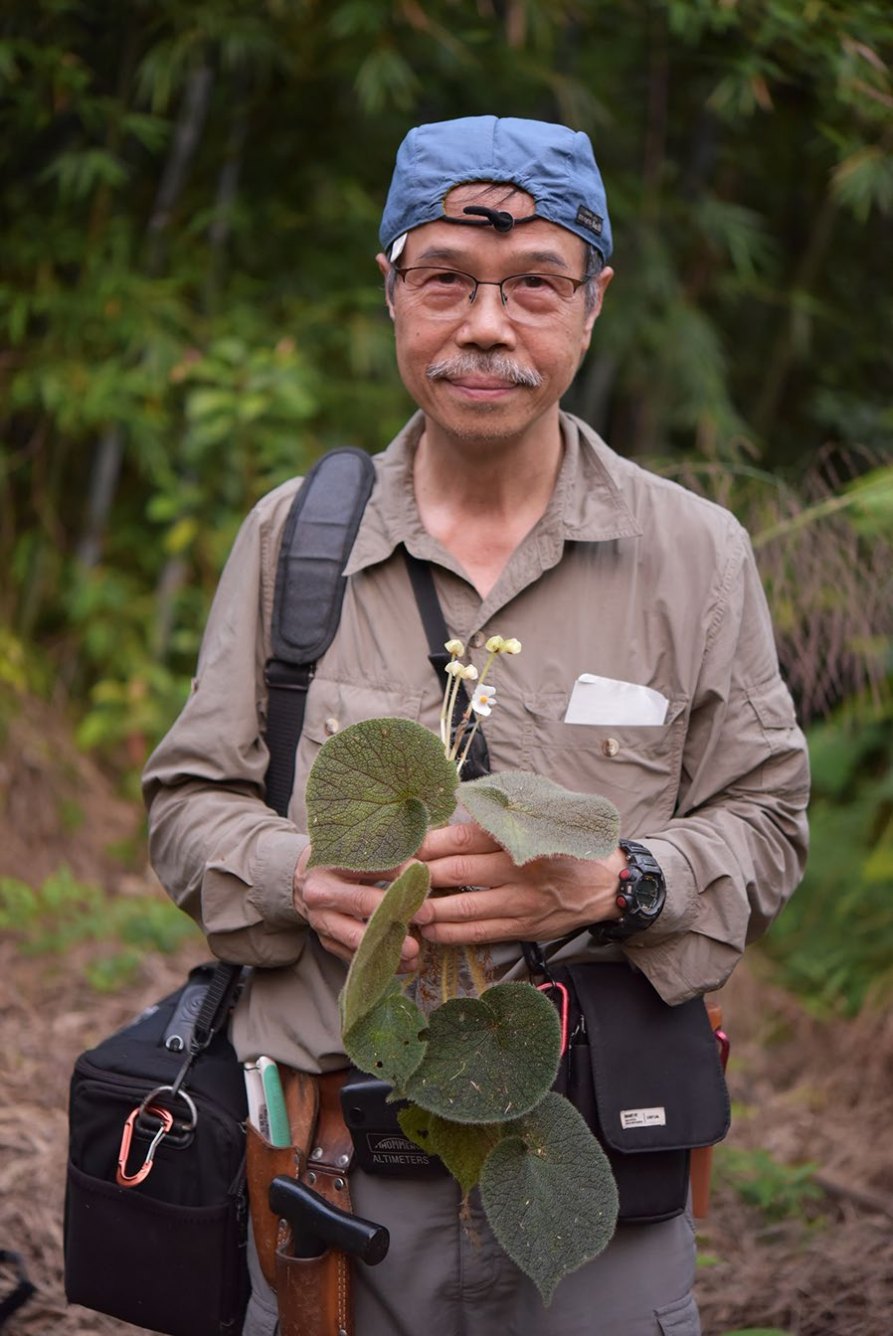
Ching-I Peng, holding *Begonia aurora* C.I Peng, Yan Liu & W.B.Xu. Photograph taken on April 18, 2016

With currently 1980 accepted species (Hughes et al. 2015), the mega-diverse genus *Begonia* L. is the fastest growing and now perhaps the fifth largest flowering plant genus (Moonlight et al. 2018), doubling from ca. 900 species in 1997 (Frodin 2004) to the current size in merely two decades. One of the major impetus of *Begonia*’s phenomenal growth in the past decades has been the passion and dedication of Dr. Ching-I Peng (Fig. 1) and his collaborations with *Begonia* researchers and enthusiasts around the world; together the effort has resulted in 81 publications and 98 new *Begonia* species (Chung 2020). Sadly, Dr. Peng died of illness on 1 May 2018 and his prolific career of *Begonia* research was ended prematurely (Chung 2020), leaving numerous unfinished works.

To continue his enduring legacy of *Begonia* research, we here report six new *Begonia* species that were discovered during Dr. Peng’s field trips to Guangxi, China. Known for its splendid limestone karst (Chung et al. 2014; Tseng et al. 2019), Guangxi also harbors the second richest *Begonia* flora of China, surpassed only by Yunnan Province (Tian et al. 2018). The addition of these six new species increases the total number of *Begonia* species in Guangxi from 86 (Dong and Liu 2019; Tong et al. 2019) to 92, with more than 1/4 of the *Begonia* species named by Dr. Peng and his collaboration with Guangxi Institute of Botany (Chung 2020). According to morphological characters and comparison with allied species, one of the six new species, *B. aurora*, might be assigned to Sect. *Platycentrum* (Klotzsch) A.DC. (sensu Moonlight et al. 2018), while the other five, viz. *B. larvata*, *B. longiornithophylla*, *B. lui*, *B. scabrifolia*, and *B. zhuoyuniae*, are allied to Sect. *Coelocentrum* Irmsch. (sensu Chung et al. 2014). Molecular phylogenetic analyses were conducted to further assure their sectional placements.

## Methods

### Morphological observations

Rhizomes of the six new species collected in the field were cultivated in the experimental greenhouse of the Biodiversity Research Center, Academia Sinica, Taipei, Taiwan. Full grown plants with flowers and fruits were used for morphological observation and comparison with morphologically similar species.

### Chromosome analysis

Root tips of *Begonia longiornithophylla* and *B. zhuoyuniae* were pretreated with 2 mM 8-hydroxyquinoline solution at 15–18 °C for about 8 h and fixed overnight in ethanol-acetic acid (3:1) below 4 °C and then macerated by an enzyme mixture containing 2% Cellulase Onozuka R-10 (Yakult Honsha, Tokyo, Japan) and 1% Pectolyase (Sigma, St. Louis, MO, USA) at about 37 °C for 1 h. Subsequently, chromosomes were stained with a 2% Giemsa solution (Merck, Darmstadt, Germany). Following Levan et al. (1964), the chromosome complements were classified based on centromere position at mitotic metaphase. Voucher specimens (*B. longiornithophylla*: *Peng* et al*. 21518* and *B. zhuoyuniae*: *Peng* et al*. 21061*) are deposited in HAST.

### Phylogenetic analyses

DNA sequences of three non-coding plastid DNA regions, *ndhA* intron, *ndhF*-*rpl32* spacer, and *rpl32*-*trnL* spacer, were used for phylogenetic analysis according to Moonlight et al. (2018), the most comprehensive phylogenetic study of *Begonia* up to date. DNA sequences of *B. scabrifolia* were generated by PCR and the remaining five new species were retrieved from full plastome sequences assembled for our ongoing phylogenomic projects of *Begonia* using the genome skimming approach (Twyford and Ness 2017). Additionally, DNA sequences of the three regions were also obtained from whole plastom sequence of *B. bamaensis* that is morphologically similar to *B. scabrifolia*.

High quality genomic DNA was extracted using the DNeasy Plant Mini kit (Qiagen, Germany). The quantity and quality of DNA were then measured by Qubit^TM^ 3.0 Fluorometer (Thermo Scientific, MA, USA) and by NanoDrop^TM^ 2000 spectrophotometer (Thermo Scientific, MA, USA), respectively. For *B. scabrifolia*, PCR amplification and DNA sequencing followed Thomas et al. (2011). For the remaining five new species and *B. bamaensis*, the genomic DNA was sent to whole genome shotgun sequencing (Illumina Hiseq, 250 bp paired-end reads) in the High Throughput Genomics Core at Biodiversity Research Center, Academia Sinica (BRCAS). The sequencing quality of the raw reads were then evaluated by FASTQC v0.11.5 (Andrews 2010). Low quality portions of the reads were trimmed and filtered out by Trimmomatic 0.36 (Bolger et al. 2014). Subsequently, the published plastome sequences of *Begonia* (Harrison et al. 2016) were used as reference to perform reference-based assembly using the option “Map to Reference” in Geneious Prime (Kearse et al. 2012) to generate a daft genome. Reads were subsequently mapped back to the draft genome to check if there were any problematic assemblies which might be resulted from some minor structural differences between our data and the references. By extracting the correct regions (as contigs) and mapping reads on them to extend these contigs, the problematic parts could be resolved. These extended contigs were mapped back to the draft sequence to correct the assembly, generating a circular genome. The completely assembled plastomes were annotated by GeSeq (Tillich et al. 2017) web tool based on plastomes of *Begonia* (Harrison et al. 2016). The start and stop codons of each gene were manually checked and adjusted under Geneious Prime. The tRNA genes were further checked by referring to the secondary structures drawn by tRNAscan-SE web server (Chan et al. 2019).

In addition to the six new species and *B. bamaensis*, DNA sequences of 80 additional Asian *Begonia* species were downloaded from NCBI (Additional file 1), including one species of Sect. *Alicida* (§*ALI*), six species of Sect. *Baryandra* A.DC. (§*BAR*), eight species of Sect. *Coelocentrum* Irmsch. (§*COE*), 16 species of Sect. *Diploclinium* (Lindl.) A.DC. (§*DIP*), one species of Sect. *Haagea* (Klotzsch) A.DC. (§*HAA*), one species of Sect. *Lauchea* (Klotzsch) A.DC. (§*LAU*), three species of Sect. *Parvibegonia* A.DC. (§*PAR*), seven species of Sect. *Petermannia* (Klotzsch) A.DC. (§*PET*), 35 species of Sect. *Platycentrum* (Klotzsch) A.DC. (§*PLA*), two species of Sect. *Reichenheimia* (Klotzsch) A.DC. (§*REI*), and *B. boisiana* Gagnep. not yet assigned to section (§*Ignota*). *Begonia rigida* Linden ex Regel of Sect. *Pritzelia* (Klotzsch) A.DC. (§*PRI*) and *B. komoensis* Irmsch. of Sect. *Tetraphilia* A.DC. (§*TET*) were included as outgroups based on Moonlight et al. (2018).

The three plastid sequences were concatenated using amas-0.93 (Borowiec 2016). Sequences were aligned using MAFFT (Katoh and Standley 2013). The final alignment is available as the Additional file 2. The maximum likelihood analyses with 1000 bootstrap resampling were conducted using RAxML-HPC (Stamatakis et al. 2008), with a gamma model of rate heterogeneity and the substitution model GTR + G+I.

## Results and discussion

### Phylogenetic analyses

**Fig. 2:**
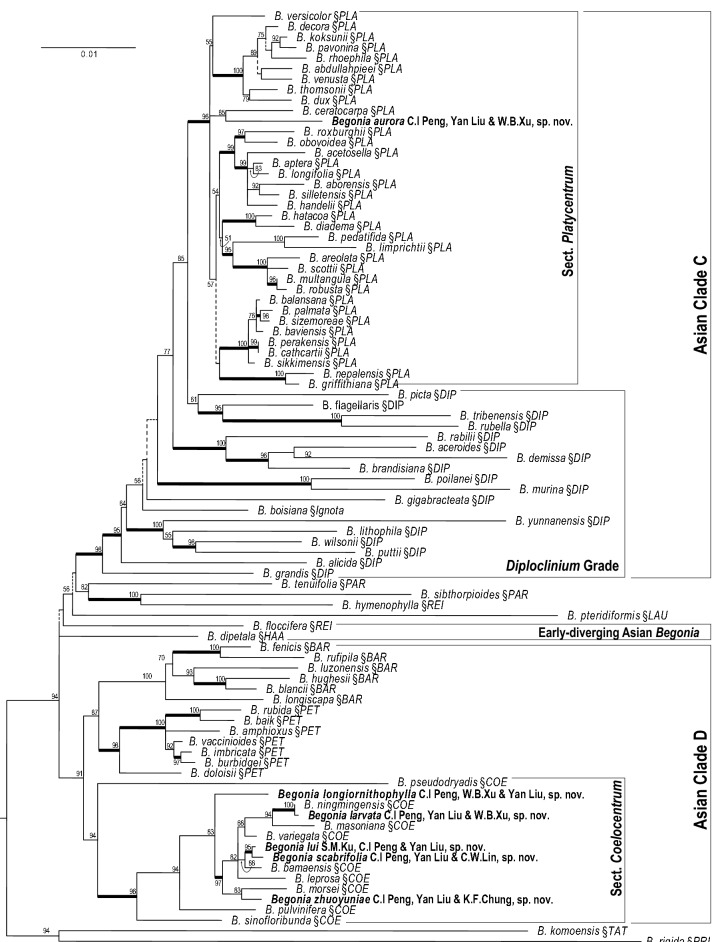
A maximum likelihood phylogram based on plastid DNA sequences of *ndhA*, *ndhF*-*rpl32*, and *rpl32*- *rpL32*-*trnL* and bootstrap support values (> 50). See text for abbreviation of section names

The alignment (Additional file 2) of the concatenated matrix of the three plastid regions contained 4532 characters [*ndhA*: 1481 bp (87 sequences); *ndhF*-*rpL32*: 1455 bp (85 sequences); *rpL32*-*trnL*: 1596 bp (87 sequences)]. The maximum likelihood tree reconstructed by RAxML-HPC and bootstrap support (BS) values are depicted in Fig. 2. The overall relationship is highly congruent with Moonlight et al. (2018), with both Sect. *Platycentrum* (BS = 96) and Sect. *Coelocentrum* (BS = 96) supported as monophyletic groups. As expected by their respective morphological characters, *B. aurora* is placed within Sect. *Platycentrum*, and the other five new species (i.e., *B. larvata*, *B. longiornithophylla*, *B. lui*, *B. scabrifolia*, and *B. zhuoyuniae*) are placed within Sect. *Coelocentrum*. Within Sect. *Platycentrum*, *B. aurora* is sister to *B. ceratocarpa* S.H.Huang & Y.M.Shui with good support (BS = 85).

### Species descriptions

**Fig. 3:**
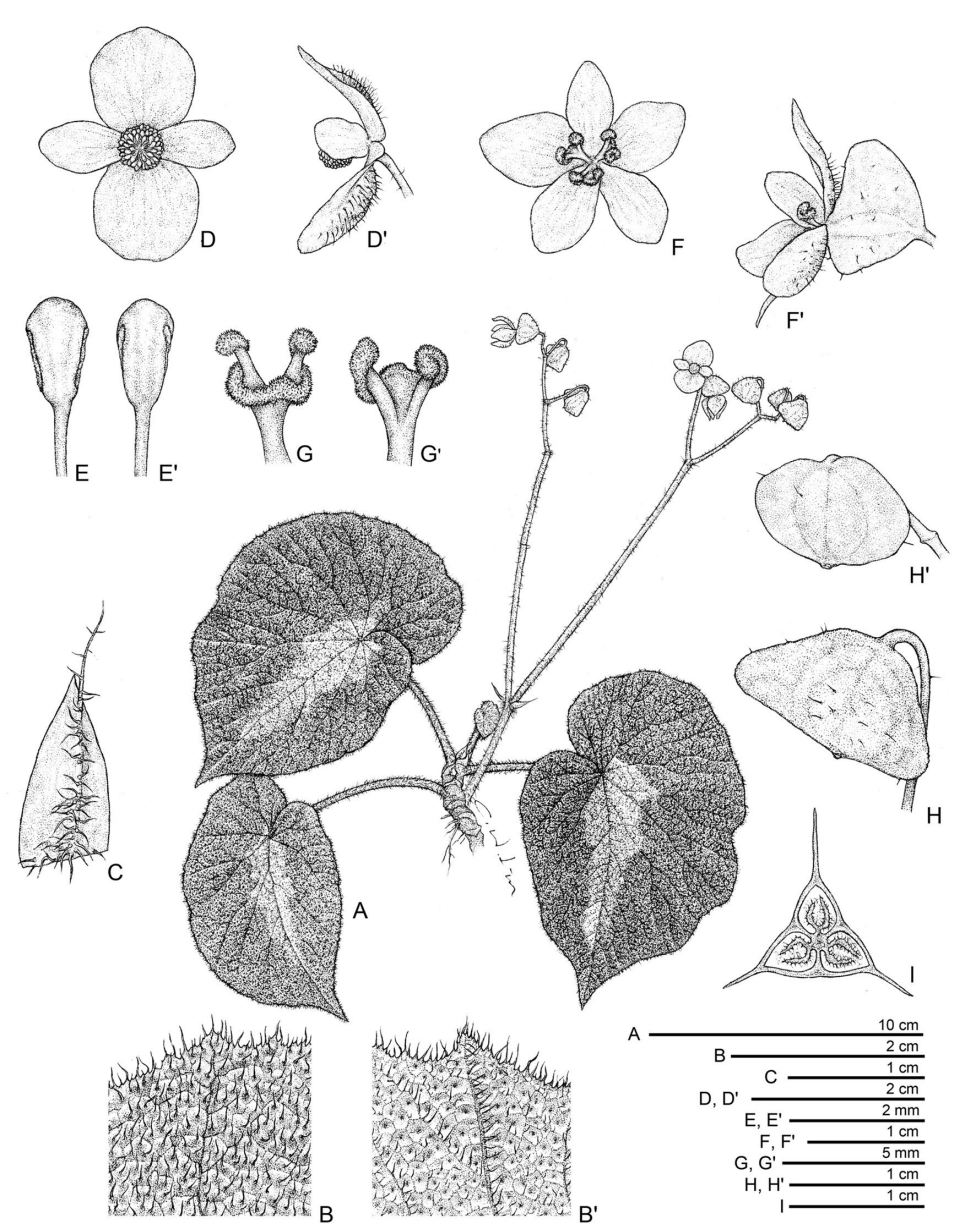
*Begonia aurora* C.I Peng, Yan Liu, W.B.Xu. **A** Habit; **B**, **B’** Portion of leaf, showing indumentum on adaxial and abaxial surfaces; **C** Stipule; **D**, **D’** Staminate flower, face view and side view; **E**, **E’** Stamen; **F**, **F’** Pistillate flower, face view and side view; **G**, **G’** Style and stigma; **H**, **H’** Fruit; **I** Ovary cross section. **A**–**C**, **H’**–**I** from *Peng* et al*. 23696*, **D**–**H** from *Peng* et al*. 24765*

**Fig. 4:**
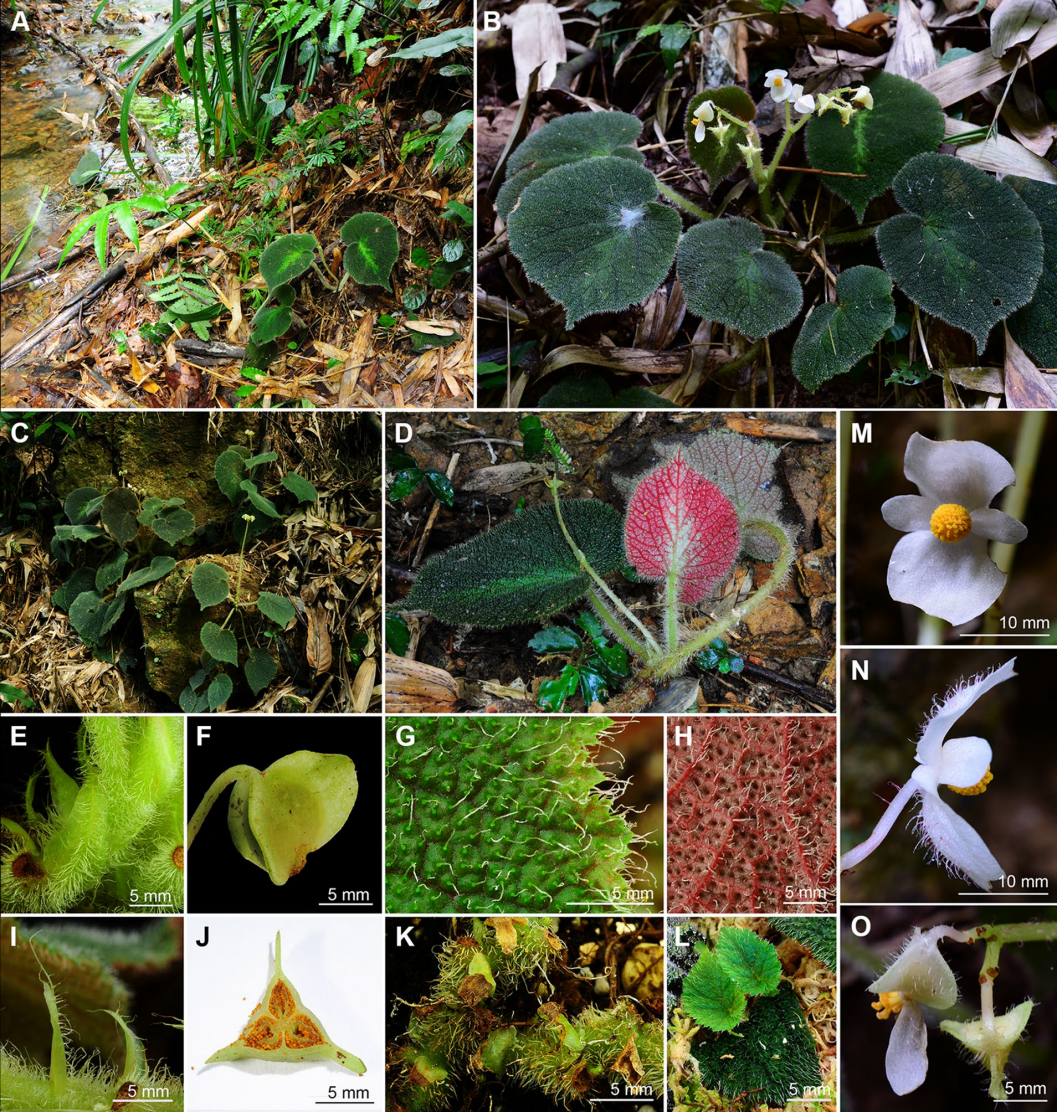
*Begonia aurora* C.I Peng, Yan Liu, W.B.Xu. **A**–**D** Habit and habitat; **E** Stipule, abaxial view; **F** Fruit; **G**, **H** Portion of leaf, showing indumentum on adaxial and abaxial surfaces; **I** Stipule, side view; **J** Ovary cross section; **K** Rhizome; **L** Propagation by leaf cutting; **M**, **N** Staminate flower, face view and side view; **O** Pistillate flower and fruit. **A**, **D**–**L** from *Peng* et al*. 23696*, **B**–**C**, **M**–**O** from *Peng* et al*. 24765*

**1.*Begonia aurora*** C.I Peng, Yan Liu & W.B.Xu, sp. nov. (Sect. *Platycentrum*) 極光秋海棠 (Figs. 3 and 4).

**Type:** CHINA. Guangxi, Fangchenggang City, Fangcheng District, Nasuo Town, 21°41’39.6”N, 108°05’39.3”E, elev. 65 m, at base of a north-facing slope beside a streamlet, fruiting and flowering, 18 April, 2016, *Ching*-*I Peng 24765* with *Kuo*-*Fang Chung*, *Wei*-*Bin Xu*, *Chia*-*Lun Hsieh* (holotype: IBK; isotypes: E, HAST-144966, K, KUN, PE).

Monoecious rhizomatous herb. **Rhizomes** creeping, to 10 cm or longer, 5 − 10 mm in diameter, internodes congested, up to 8 mm long, light green, densely villous. **Stipules** persistent, pale green, ovate, ca. 15 mm long, 6–7 mm wide, herbaceous, strongly keeled, densely velutinous along midrib abaxially, margin entire, apex aristate, arista ca. 7 mm long. **Leaves** alternate; petiole terete, pale green, 5 − 15 cm long, 3–5 mm thick, densely white villous; leaf blade asymmetric, oblique, widely ovate, 6 − 17 cm long, 4–12 cm wide, broader side 2.5–8.5 cm wide, basal lobes cordate, 1.8–6 cm long, apex acuminate, margin denticulate and densely white villous; leaves chartaceous, adaxially deep green to dark viridian, often embellished with lime green zone around midrib; venation reddish and impressed, densely covered by small raised cones between veins, giving the lamina a rugose appearance, each cone topped by a single white villous hair ca. 1.5 mm long; abaxially purplish red (rarely green), sometimes with a pale green zone along midrib, white villous on all veins; venation palmate, midrib distinct, with ca. 3 secondary veins on each side, tertiary veins reddish, percurrent or reticulate. **Inflorescences** bisexual, axillary, dichasial cymes arising directly from rhizome, branched 2–4 times; peduncle pale green, 3–10 cm long, pilose; bracts pale green to pinkish, hyaline, thin chartaceous, those at basal node of inflorescence ovate, 1–1.8 cm long, 4–5 mm wide, margin entire; bracts at summit of inflorescence similar but smaller. **Staminate flowers:** pedicel 7–17 mm long, sparsely pilose, tepals 4, white, outer 2 widely obovate to suborbicular, 10–15 mm long, 10–12 mm wide, abaxially pilose, inner 2 elliptic to oblanceolate, ca. 11 mm long, 6 mm wide, apex obtuse to rounded; androecium actinomorphic, ca. 5 mm across; stamens yellow, ca. 90; filaments fused on a short stalk; anthers obovate, ca. 2 mm long, 2-locular, apex rounded, subequal to filaments. **Pistillate flowers:** pedicel ca. 11 mm long, sparsely pilose; tepals 5, white, ovate, 6–12 mm long, 6–9 mm wide, apex obtuse or rounded, outer 3 abaxially pilose; ovary pale green, body trigonous-ellipsoid, ca. 9 mm long, 3 mm thick (wings excluded), pilose; 3-winged, wings unequal, abaxial wing triangular or crescent-shaped, margin entire, ca. 3 mm high, apex rounded or slightly pointed at summit; 3-locular, placentation axile, bilamellate; styles 3, shortly fused at base, yellow, ca. 3.5 mm long, stigma spirally twisted. **Capsules** pendent, pedicel 10–16 mm long, tepals deciduous; body trigonous-ellipsoid, 8–12 mm long, 5–6 mm thick (wings excluded), greenish when fresh; abaxial wing 4–6 mm high, lateral wings 3–4 mm high.

### Distribution and ecology

*Begonia aurora* is known only from the type locality where less than 50 plants were seen. Plants grow on a slope of a shaded gully in a mixed forest of broadleaved woods and bamboo plantation.

### Phenology

Flowering from March to April, fruiting from April to June.

### Etymology

The species epithet refers to the lighter green patch around the midrib of the leaves resembling aurora, the polar lights.

### Additional specimen examined (paratype)

CHINA. Guangxi, Fangchenggang City, Fengcheng District, Nasuo Town, elev. 53 m, 31 May 2012, *Ching*-*I Peng 23696* with *Wei*-*Hsin Hu*, *Yu*-*Song Huang*, and *Shui*-*Song Mo* (HAST-144551).

### Notes

Our phylogenetic analyses placed *Begonia aurora* in Sect. *Platycentrum* sensu Moonlight et al. (2018) with strong support (Fig. 2). Amongst the other 35 sampled species of the section, *B. aurora* is sister to *B. ceratocarpa* (BS = 85). Morphologically, *B. aurora* is somewhat similar to *B. versicolor* Irmsch. in its hairy leaves with small cone-like structures on leaf blade. Nevertheless, *B. aurora* can be easily distinguished from *B. versicolor* by its dark green (vs. variegated or pure green) leaves, white (vs. pink) tepals, and 3-locular (vs. 2-locular) ovary.

**Fig. 5:**
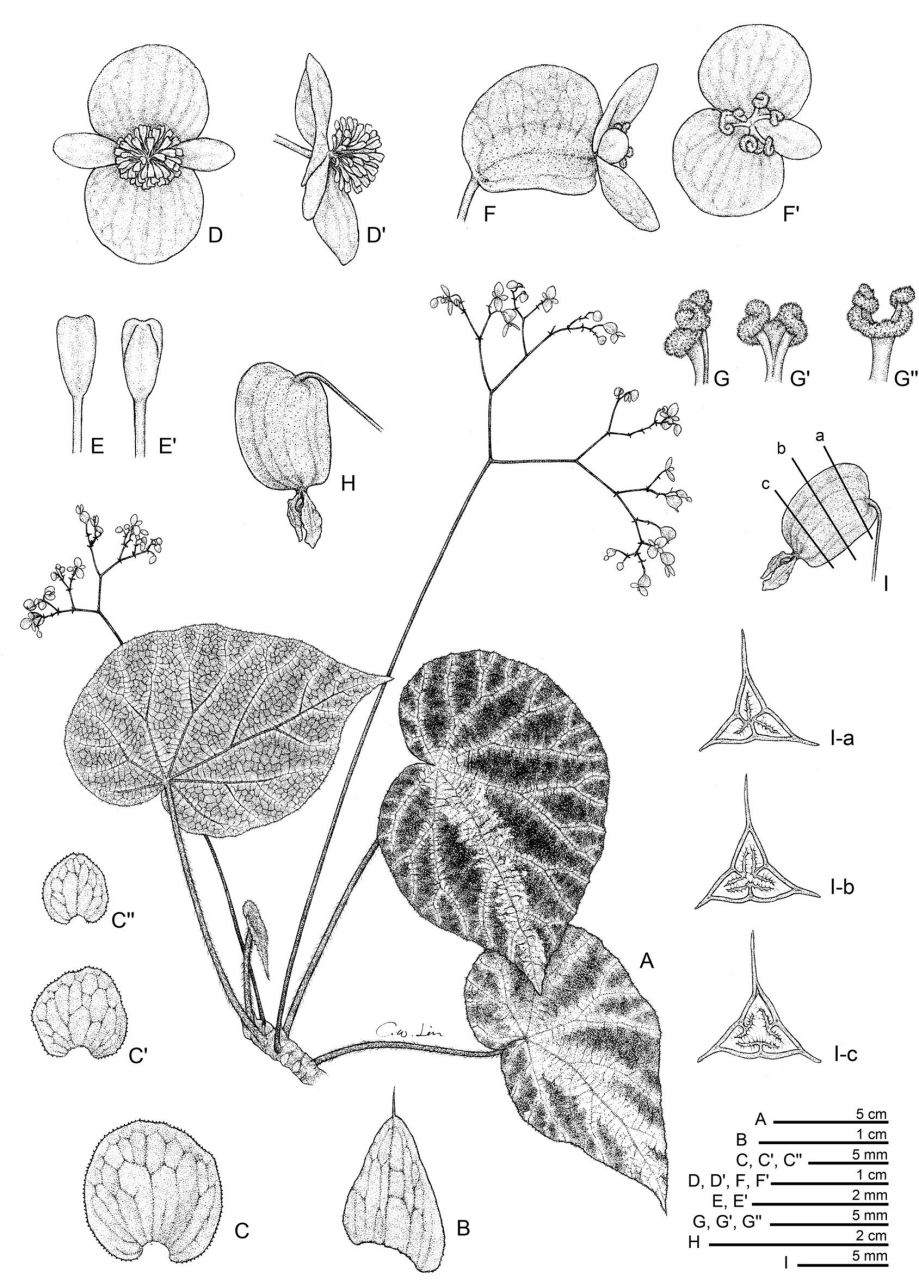
*Begonia larvata* C.I Peng, Yan Liu & W.B.Xu. **A** Habit; **B** Stipule; **C**, **C’**, **C’’** Bracts, lower to upper; **D, D’** Staminate flower, face view and side view; **E, E’** Stamen, dorsal and ventral views; **F**, **F’** Pistillate flower, side views, face view; **G**, **G’**, **G’’** Style and stigmatic band, side, dorsal and ventral views; **H** Capsule; **IA**–**IC** Serial cross section of an immature capsule. All from *Peng* et al*. 24372* (HAST)

**Fig. 6:**
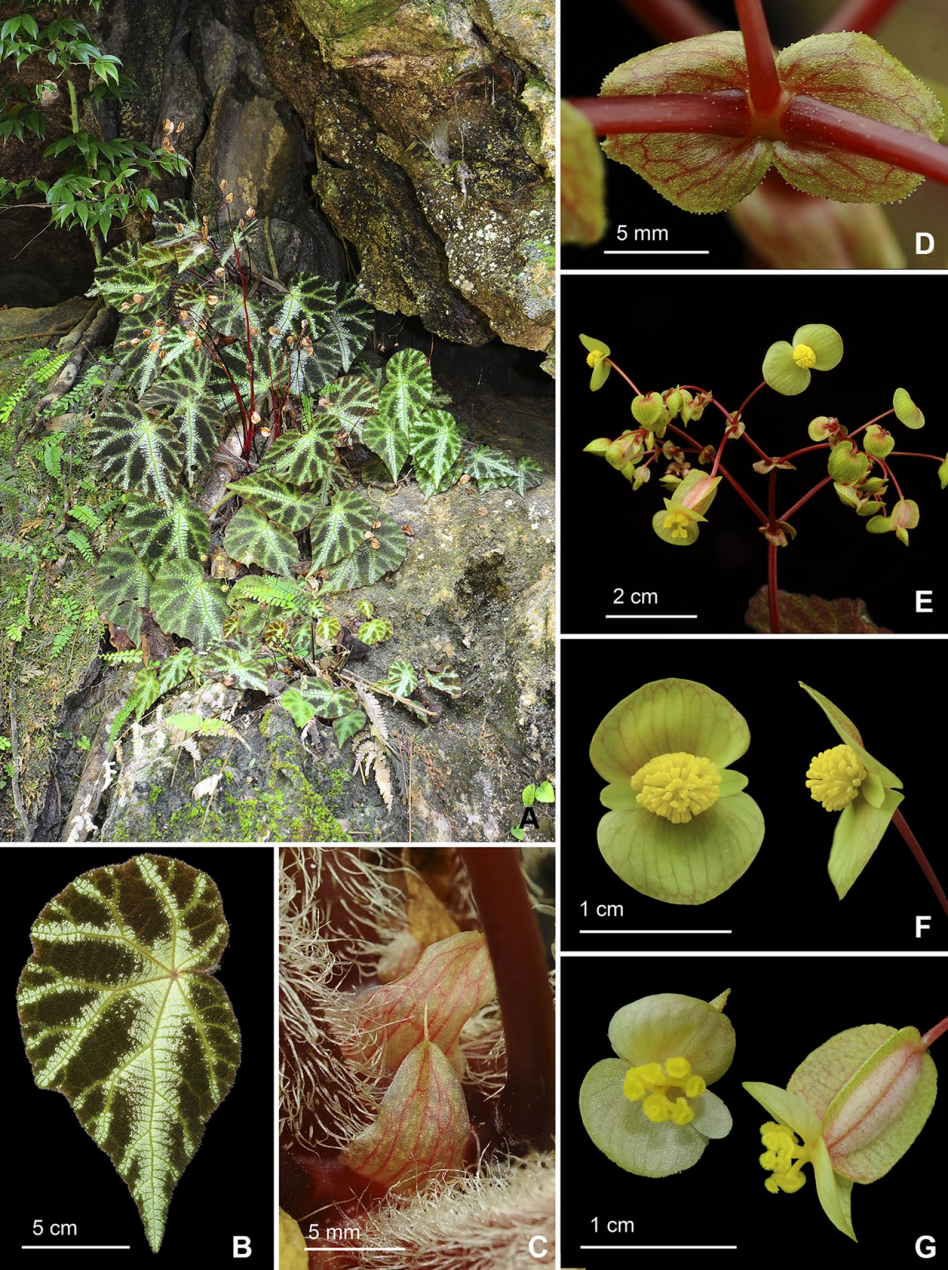
*Begonia larvata* C.I Peng, Yan Liu & W.B.Xu. **A** Habitat and habit; **B** Leaf adaxial view; **C** Stipules; **D** Bracts; **E** Inflorescence, showing 2 tepals flowers; **F** Staminate flowers; **G** Pistillate flowers. All from *Peng* et al*. 24372* (HAST)

**2.*Begonia larvata*** C.I Peng, Yan Liu & W.B.Xu, sp. nov. (Sect. *Coelocentrum*) 果子狸秋海棠 (Figs. 5 and 6).

**Type:** CHINA. Guangxi, Chongzuo City, Jiangzhou District, Zuozhou Town, Guanghe Village, Pairu Tun, elev. ca. 100 m, 15 June 2014, *Peng* et al*. 24372* with Wei-bin Xu (holotype: IBK; isotype: HAST-138465).

Monoecious rhizomatous herb. **Rhizomes** stout, creeping, to 15 cm or longer, 10–18 mm thick, internodes 5–10 mm long, glabrous but villous at the base of petiole. **Stipules** persistent, yellowish green with red veins, triangular-ovate, 8–15 mm long, 7–12 mm wide, strongly keeled, glabrous, margin entire, apex aristate, arista ca. 4 mm long. **Leaves** alternate; petiole terete, reddish, (9 −)14 − 22 cm long, 4–6 mm thick, densely white villous; leaf blade asymmetric, oblique, ovate to widely ovate, 13 − 22 cm long, 9–16 cm wide, broad side 5–10 cm wide, basal lobes cordate, 3.8–7 cm long, apex acuminate, margin denticulate and densely puberulous, hairs white or pale magenta; leaf thick chartaceous, adaxially deep olive-green to dark viridian, emerald to lime green zone and embellish with crushing silvery white spots along primary and secondary veins, midrib veins forming a widely silvery white zone; surface densely small raised cones topped, a top by a hair, hair white to magenta, give a wrinkled texture; abaxially pale green, purplish red between primary and secondary veins, pilose on all veins; venation basally ca. 7 palmate, midrib distinct, ca. 3 secondary veins on each side, tertiary veins reddish, lateral through connection between with other basal veins, minor veins reticulate. **Inflorescences** axillary, dichasial cymes or diffusely thyrsoid, arising directly from rhizome, branched 5–8 times; peduncle crimson, 17–32 cm long, glabrous; bracts usually persistent, pale yellow-green with reddish veins, orbicular to widely ovate, first pair ca. 8 mm across, glabrous, margin entire with sessile glands, bracts of upper inflorescence similar but smaller. **Staminate flower:** pedicel 0.8–3 cm long, glabrous, tepals 4 (sometimes 2), glabrous, outer 2 very widely ovate to orbicular, 5–10 mm long, 8–12 mm wide, yellowish green with red veins, sometimes reddish toward the base, inner 2 obovate, yellowish green, ca. 4 mm long, 2 mm wide; androecium actinomorphic, 3.5–6 mm across; stamens golden yellow, 65–80; filaments fused at base; anthers obovate, ca. 1 mm long, 2-locular, apex truncate or retuse, more or less equal at filaments. **Pistillate flower:** pedicel 1–2.3 cm long, glabrous, tepals 3 (sometimes 2), glabrous, outer 2 suborbicular or broadly ovate, yellowish green with red veins, 7–10 mm long, 8–11 mm wide, inner 1 obovate, yellowish green, ca. 3 mm long, 2 mm wide; ovary trigonous-ellipsoid, 7–11 mm long, 3.5–5 mm thick (wings excluded), yellow-green, redness, sparsely glands; 3-winged, wings unequal, yellowish green with red veins, sparsely glands, lateral wings narrower, narrowly crescent-shaped to trapezium, 2–3 mm high, abaxial wing crescent-shaped, ca. 4 mm high, margin entire; styles 3, fused at base, golden yellow, ca. 3.5 mm long, stigma spirally twisted. **Capsule:** tepals persistent; capsule trigonous-ellipsoid, 8–14 mm long, 4–5 mm thick (wings excluded), greenish or reddish when fresh; wings unequal, lateral wings ca. 4 mm high, abaxial wing crescent-shaped, ca. 5 mm high.

### Distribution and ecology

*Begonia larvata* is only known from the Zuozhou Town, Jiangzhou District, Chongzuo City, Guangxi, growing on semi-shaded limestone cliff surfaces or steep slopes, at 125–165 m in elevation.

### Etymology

The species epithet ‘larvata’ (masked), refers to the leaf variegation that resembles the facial mark of masked palm civet (*Paguma larvata*).

### Additional specimen examined (paratype)

CHINA. Guangxi, Chongzuo City, Jiangzhou District, Zuozhou Town, Guanghe Village, Pairu Tun, 22°35’28.589”N, 107°25’40.007”E, elev. 144 m, 3 December 2019, *Wei*-*Bin Xu 14146* with *Li*–*Na Dong*, *Yu*-*Hsin Tseng* (HAST-144947).

### Notes

*Begonia larvata* is similar to *B. pengii* S.M.Ku & Yan Liu (Ku et al. 2008) but can be easily distinguished from the latter by its basifixed (vs. peltate) leaves, persistent (vs. caduceus) bracts, glabrous (vs. pilose or hispid-villous) peduncles, yellowish green (vs. pinkish or whitish) tepals, and glabrous (vs. pilose or hispid-villous) ovaries. *Begonia larvata* also resembles *B. locii* C.I Peng, C.W.Lin & H.Q.Nguyen (Peng et al. 2015), differing from the latter by its entire bract (vs. denticulate) margins, glabrous (vs. tomentose to glabrous) peduncles, yellowish green (vs. white to pinkish) tepals, and glabrous (vs. hirsute) ovary. A comparison of the salient characters of the three species is shown in Table 1.

**Table 1:** Comparison of *Begonia larvata* with *B. locii* and *B. pengii*

	*Begonia larvata*	*B. locii* (Peng et al. 2015)	*B. pengii* (Ku et al. 2008)
Leaf			
Attachment	Basifixed	Basifixed	Peltate
Stipule	Triangular-ovate, glabrous, margin entire or sparsely ciliate-dentate	Triangular-ovate, abaxially sparsely velutinous along midrib, margin entire or sparsely ciliate-dentate	Narrowly triangular-ovate, glabrous, margin eciliate
Bracts			
Margin	Entire and glandular	Denticulate and glandular	Denticulate and ciliate
Inflorescence			
Peduncle	Glabrous	Tomentose to glabrous	Pilose or hispid-villous
Tepals color	Yellowish green with red veins	White to pinkish	Pinkish or white
♂ Flower			
Androecium	Actinomorphic	Zygomorphic	Zygomorphic
Stamen numbers	65–80	35–60	30–75
♀ Flower			
Ovary	Glabrous	Hirsute	Pilose or villous-pilose
Capsule			
Body size (mm)	8–13 × 4–5	10–15 × 4–5	18–25 × 6–13
Width of lateral wings (mm)	ca. 4	4–6	2–3
Width of abaxial wing (mm)	ca. 5	6–7	7–11
Tepals	Persistent	Caducous	Caducous

**Fig. 7:**
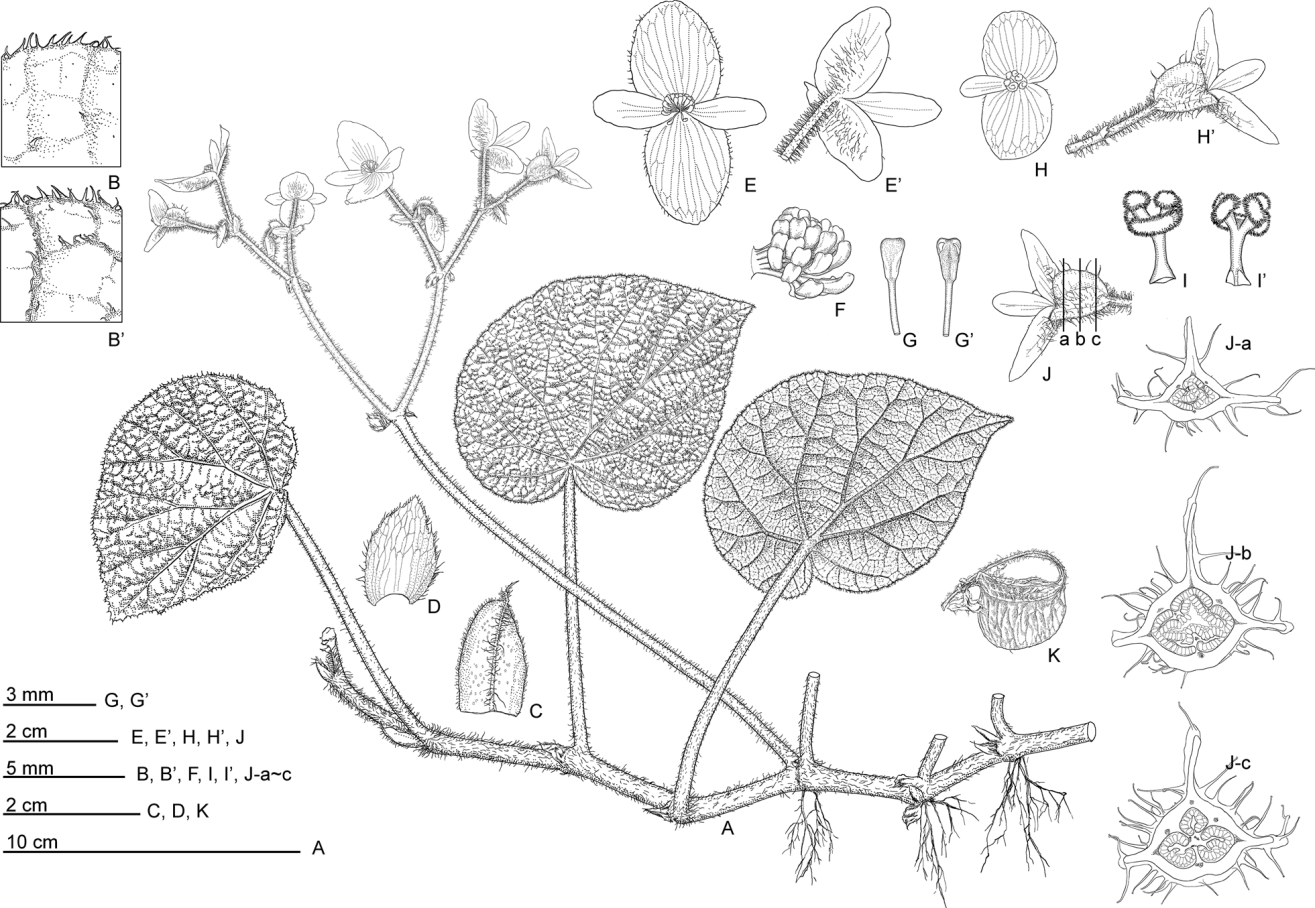
*Begonia longiornithophylla* C.I Peng, W.B.Xu & Yan Liu. **A** Habit; **B**, **B’** Portion of leaf, showing indumentum on adaxial and abaxial surfaces; **C** Stipule; **D** Bract; **E**, **E’** Staminate flower, face view and back view showing indumentum; **F** Androecium, side view; **G**, **G’** Stamen; **H**, **H’** Pistillate flower, face view and back view; **I**, **I’** Style and Stigma; **J-A**–**J-C** Serial cross sections of ovary; **K** Fruit. All from *Peng* et al*. 21518* (HAST)

**Fig. 8:**
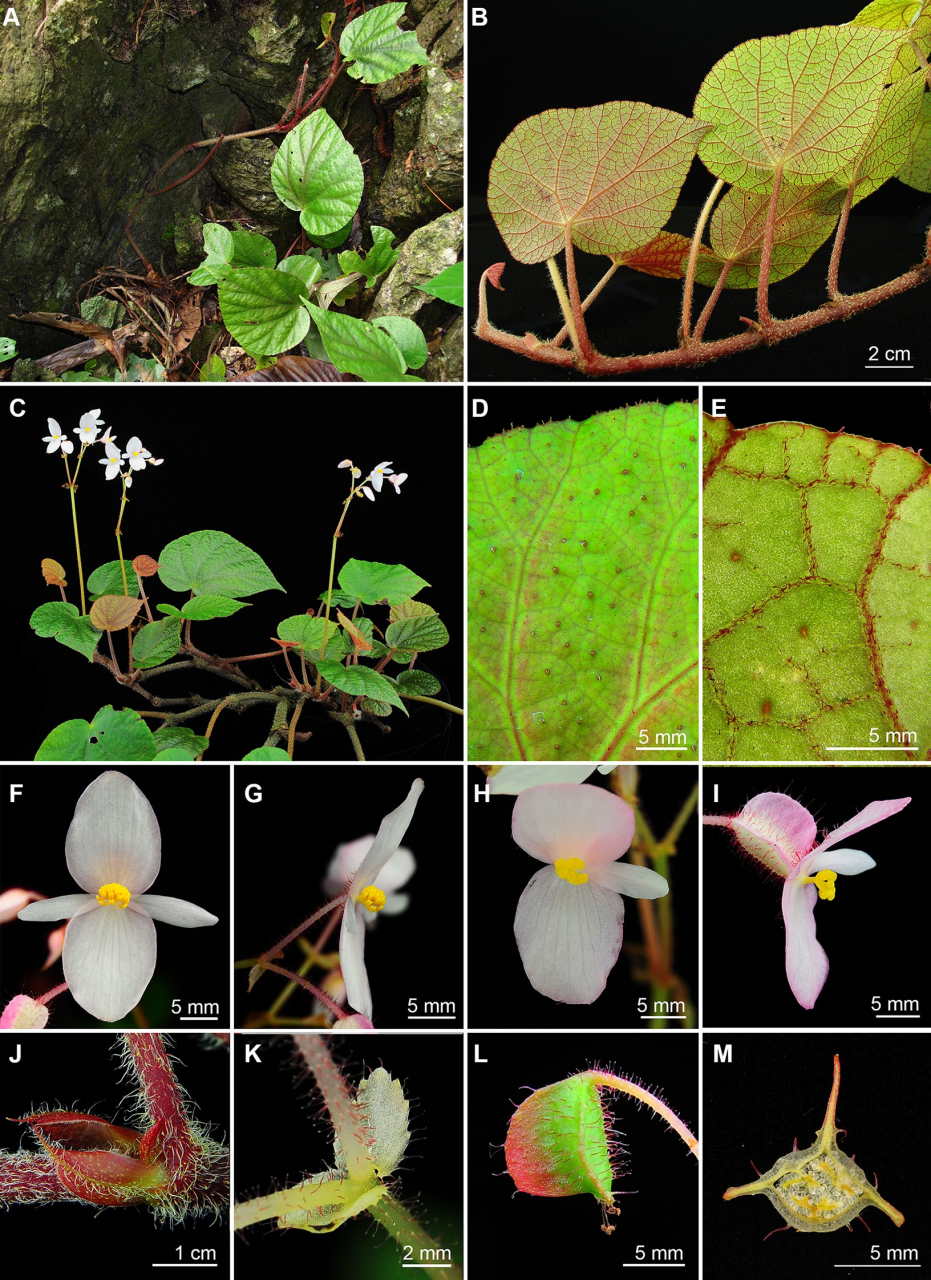
*Begonia longiornithophylla* C.I Peng, W.B.Xu & Yan Liu. **A** Habitat and habit; **B** Habit, showing rhizome and leaf abaxial surface; **C** Cultivated plant at anthesis; **D** Leaf adaxial surface; **E** Leaf abaxial surface; **F**, **G** Staminate flower, face view and side view; **H**, **I** Pistillate flower, face view and side view; **J** Stipules and petiole, showing indumentum; **K** Bracts, showing glandular hairs on peduncle and pedicels; **L** Young fruit; **M** Cross section of ovary. All from *Peng* et al*. 21518* (HAST)

**3.*Begonia longiornithophylla*** C.I Peng, W.B.Xu & Yan Liu, sp. nov. (Sect. *Coelocentrum*) 長莖鳥葉秋海棠 (Figs. 7 and 8).

**Type**: CHINA, Guangxi, Chongzuo Shi, Daxin County, Xialei Town, Tiandeng Tun, on rocky forest floor, 22°52’19”N, 106°43’31”E, elev. ca. 550 m, plant collected on 23 June 2008, type specimens (in flowers) pressed from plants cultivated in the experimental greenhouse, Academia Sinica, Taiwan, *Ching*-*I Peng 21518*-*A* with *Shin*-*Ming Ku, Chih*-*Kai Yang, Wei*-*Bin Xu, Bo Pan & Yun*-*Fei Deng* (holotype: IBK; isotypes: E, HAST-144967, K, KUN, PE).

Monoecious rhizomatous herb. **Rhizomes** much elongate, to 50 cm long, 6–12 mm across, internodes (1–)3–9 cm long, purple red, pilose to tomentose. **Stipules** persistent, red, herbaceous, ovate, ca. 2 cm long, 8 mm wide, strongly-keeled, apex aristate, arista ca. 3 mm long, villous along midrib, margin ciliate. **Leaves** alternate; petioles terete, 3–21 cm long, 5 mm across, red-brown, villous to tomentose; leaf blade asymmetric, ovate, 5.5–12 cm long, 3.5–8 cm wide, apex acuminate, base strongly obliquely cordate, subcoriaceous, adaxially green, scabrid and glandular punctate, abaxially red-scabrous on veins, margin crenulate and ciliate; venation palmate. **Inflorescences** axillary, arising directly from rhizome, cymes dichasial, branched 2–3 times, protandrous; peduncle 8–20 cm long, 3 mm across, glandular-pilose; bracts persistent, ovate, 10 mm long, 7 mm wide, light yellow green or somewhat with reddish veins, margin serrate, with hair on the apex of each tooth. **Staminate flower**: pedicel ca. 1.8 cm, glandular-pilose, tepals 4, outer 2 elliptic, 15–18 mm long, 12–15 mm wide, pinkish-white, abaxially red-pilose, margin sparsely ciliate, inner 2 elliptic, ca. 12 mm long, 3 mm wide, white; androecium zygomorphic, ca. 4 mm across, stamens ca. 32, filaments fused at base, obovate, 2-locular, connective apex retuse. **Pistillate flower**: pedicel ca. 18 mm long, glandular-pilose, tepals 3, outer 2 elliptic to sub-orbicular, 10–15 mm long, ca. 13 mm wide, pinkish-white, inner 1 oblanceolate, ca. 12 mm long, 4 mm wide, white, ovary trigonous-ellipsoid, 7–9 mm long, 3 mm thick (wings excluded), glandular-pilose, 1-locular, placenta parietal, 3-winged; wings unequal, abaxial wing crescent-shaped, ca. 4 mm high, lateral wings 2 mm high, pinkish; styles 3, fused at base, yellow, 5 mm long, stigma spirally twisted. **Capsules** trigonous-ellipsoid, ca. 13 mm long, 5 mm thick (wings excluded), style and stigma persistent; abaxial wing ca. 5 mm high, lateral wings 3 mm high.

### Chromosome cytology

**Fig. 9:**
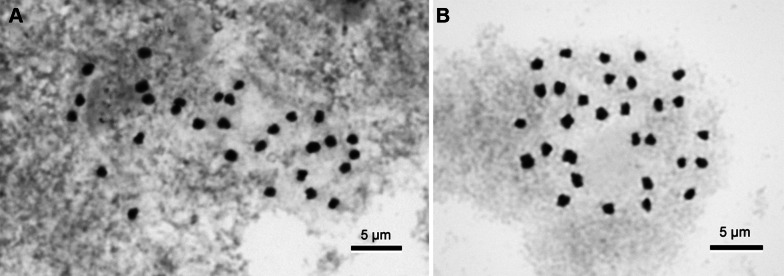
Somatic chromosomes at metaphase. **A***Begonia longiornithophylla* (2*n* = 30, from *C.*-*I Peng* et al. *21518*, HAST). **B***Begonia zhuoyuniae* (2*n* = 30, from *C.*-*I Peng* et al. *20737*, HAST). Scale bars = 5 µm

Somatic chromosomes at metaphase of *Begonia longiornithophylla* were counted as 2*n* = 30 (Fig. 9a), identical to the majority of species of Sect. *Coelocentrum* (Chung et al. 2014; Han et al. 2018). The length of chromosomes varied from ca. 1.1 to 1.6 µm long. Although several longer chromosomes were metacentric and/or submetacentric, the centromere positions of most chromosomes could not be determined. Satellites were not observed.

### Distribution and ecology

Southwestern Guangxi, China. On forest floor, creeping on limestone rocks or cliffs in broadleaf forest.

### Phenology

Flowering from February to May. Fruiting from May to July.

### Etymology

The species epithet refers to its resemblance to *Begonia ornithophylla* Irmsch., distinct from the latter by its elongated rhizomes.

### Additional specimen examined (paratype)

CHINA, Guangxi, Chongzuo City, Daxin Coumty, Xialei Town, Tiandeng Tun, on rocky forest floor, 22°52’19”N, 106°43’31”E, elev. ca. 550 m, 23 June 2008, *Ching*-*I Peng 21518* with *Shin*-*Ming Ku, Chih*-*Kai Yang, Wei*-*Bin Xu, Bo Pan & Yun*-*Fei Deng* (HAST-140791).

### Notes

*Begonia longiornithophylla* somewhat resembles *B. ornithophylla* in the leaves and *B. auritistipula* in its elongated rhizomes; the new species can be easily distinguished from the two species by several characters such as densely villous and elongated rhizomes, boat-shaped stipules, glandular-pilose peduncles, red-pilose on tepals and ovaries. A detailed comparison of the three species is provided in Table 2.

**Table 2:** Comparison of *Begonia longiornithophylla* with *B. ornithophylla* and *B. auritistipula*

	*Begonia longiornithophylla*	*B. ornithophylla*	*B. auritistipula*
Rhizome	Elongated, 6–12 mm thick, internodes 3–9 cm long, densely villous	Not elongated, 5–12 mm thick, internodes 0.6–1.5 cm long, glabrous	Elongated, 3–6 mm thick, internodes 2–6 cm long, sparesly hirsute-villous
Stipule	Red, boat-shaped, strongly keeled, villous along midrib, margin ciliate	Yellow-green with reddish veins, triangular, keeled, sparsely villous along midrib, margin entire	Yellow-green with reddish veins, broadly ovate, base obliquely auriculate, margin entire
Petiole	Villous to tomentose	Villous to tomentose	Reflexed hirsute
Leaf			
Adaxial surface	Scabrid and glandular punctate	Scabrid and glandular punctate	Setulose
Abaxial surface	Red-scabrous on veins	Villous on veins	Hirsute-pilose on veins
Texture	Subcoriaceous	Subcoriaceous	Papery
Margin	Crenulate and ciliate	Coarsely serrate or subentire	Serrulate and ciliate
Maculation	Lacking	Lacking	Brownish between primary veins
Inflorescence			
Peduncle	Glandular-pilose	Pilose	Glabrous
Abaxial surface of outer tepals	Red-pilose	Glabrous or nearly so	Glabrous or nearly so

**Fig. 10:**
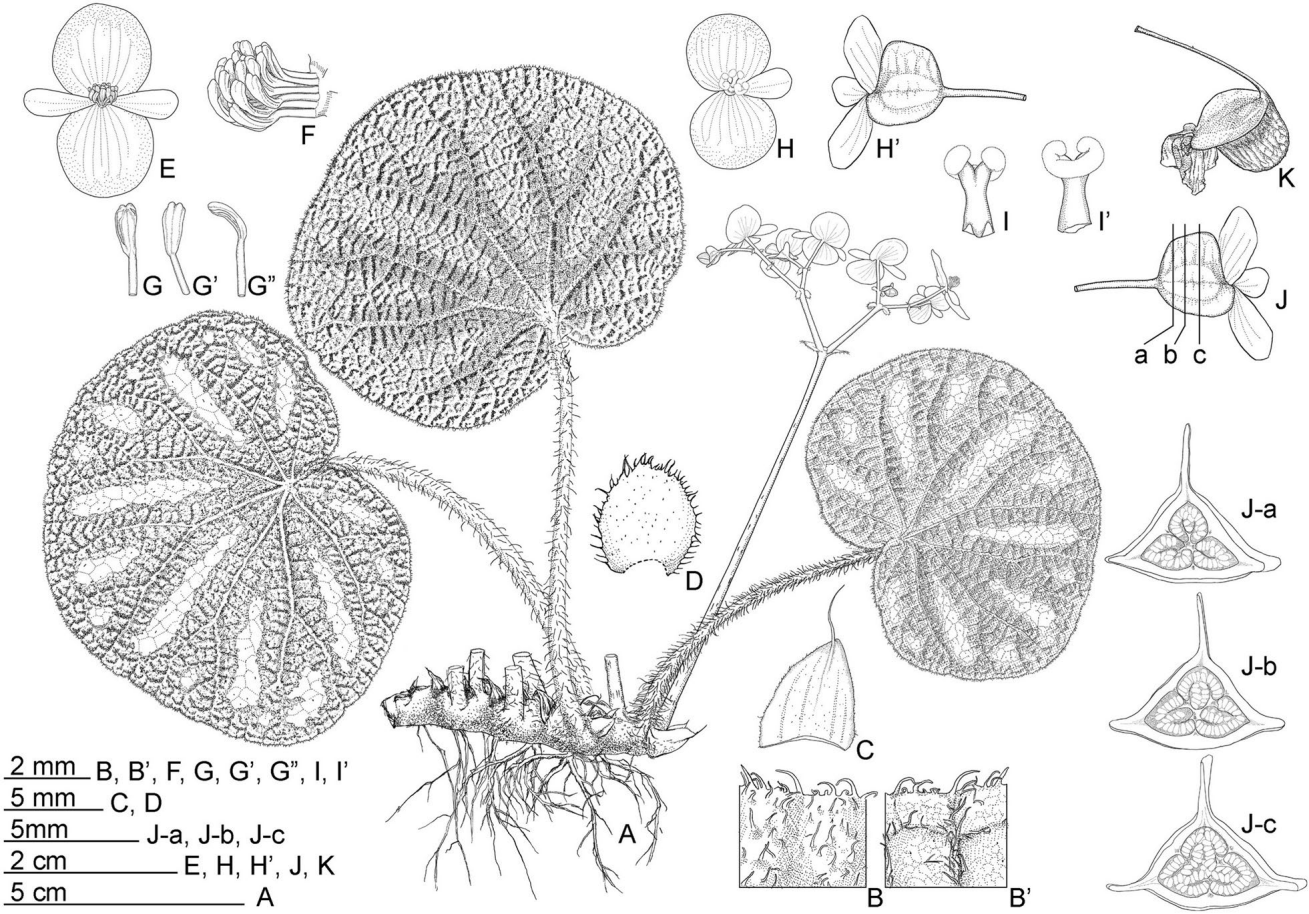
*Begonia lui* S.M.Ku, C.I Peng & Yan Liu. **A** Habit; **B** Leaf adaxial surface; **B’** Leaf abaxial surface; **C** Stipule; **D** Bract; **E** Staminate flower, face view; **F** Androecium, side view; **G**, **G’**, **G”** Stamens; **H**, **H’**, Pistillate flower; **I**, **I’** Stigmas; **J-A**–**J-C** Serial cross section of ovary; **K** Capsule. All from *Peng* et al*. 21112*

**Fig. 11:**
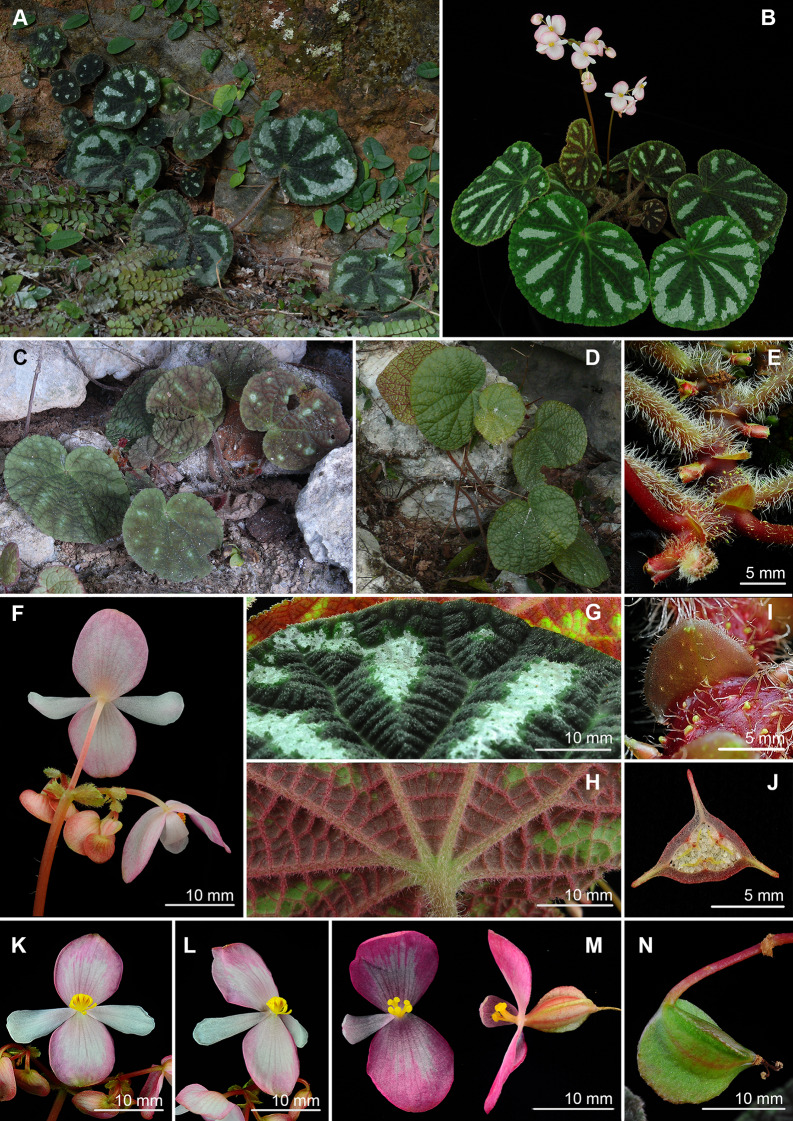
*Begonia lui* S.M.Ku, C.I Peng & Yan Liu. **A**, **C**, **D** Habitat and habit, also showing the variation of maculation on leaves among individuals; **B** Plant at anthesis; **E** Rhizome; **F** Inflorescences and bracts; **G** Leaf adaxial surface; **H** Leaf abaxial surface; **I** Stipule; **J** Ovary cross section; **K**, **L** Staminate flowers; **M** Pistillate flower; **N** Fruit. All from *Peng* et al*. 21112*

**4.*Begonia lui*** S.M.Ku, C.I Peng & Yan Liu, sp. nov. (Sect. *Coelocentrum*) 陆氏秋海棠 (Figs. 10 and 11).

*Begonia bonii* var. *remotisetulosa* Y.M.Shui & W.H.Chen, Acta Bot. Yunnan. 27(4): 360. 2005, *pro. part.* [*S.P. Ko 55623* (IBSC)].

*Begonia koi* S.M.Ku et al., sp. nov. ined. in Ku, Systematics of *Begonia* sect. *Coelocentrum* (Begoniaceae) of China. 2006.

**Type:** CHINA, Guangxi, Baise City, Jingxi County, Wuping Town, Yixing Village, Pai Lin Tun, Baiyan Cave, elev. 730 m, 23°09’28”N, 106°35’4”E, on limestone hill, plant collected on 22 May 2007, type specimens (in flowers) pressed from plants cultivated in the experimental greenhouse, Academia Sinica, Taiwan, *Ching*-*I Peng 21112*-*A* with *Yan Liu*, *Hai*-*Shan Gao*, *Kuo*-*Fang Chung*, *Ming*-*Chao Yu*, and *Lu*-*Shi Nian* (holotype: IBK; isotype: HAST-144968).

Monoecious rhizomatous herb. **Rhizomes** 5–8 mm in diameter, internodes 4–20 mm long, densely villous. **Stipules** generally persistent, ovate-triangular, 8 mm wide, 5 mm tall, margin ciliate. **Leaves** alternate; petiole 4.5–13 cm long, 2–3 mm cross, villous; leaf blade asymmetric, broadly ovate or suborbicular, 7.5–12 cm long, 6–9 cm wide, thickly chartaceous, base strongly oblique-cordate, adaxially slightly rugose, deep green, with white maculation in intercostal areas, moderately densely setulose, abaxially tomentose along veins and veinlets, margin crenulate and irregularly denticulate, ciliate, apex obtuse. **Inflorescences** axillary, dichasial cymes, arising directly from rhizome; peduncle 11–16 cm long, sparsely pilose; bracts ovate, 5–10 mm long, ca. 4 mm wide, fimbriate. **Staminate flower**: pedicel ca. 18 mm long; tepals 4, outer 2 suborbicular, ca. 13 mm in diameter, pinkish white, glabrous or nearly so, inner 2 oblanceolate, ca. 10 mm long, 4 mm wide, white; androecium zygomorphic, stamens ca. 20, filaments nearly free. **Pistillate flower:** pedicel 8–12 mm long; tepals 3, outer 2 suborbicular, ca. 10–17 mm in diameter. glabrous or nearly so, pinkish white, inner 1 oblong-obovate, ca. 12 mm long, ca. 5 mm wide, white; ovary ellipsoid, 7–10 mm long, 3–4 mm thick (wings excluded), 1-locular, placentae parietal, yellowish green to pinkish, nearly glabrous, 3-winged; wings unequal, yellowish green to pinkish, abaxial wing crescent-shaped, ca. 4 mm high, lateral wings 2, narrowly crescent-shaped, ca. 2 mm high; styles 3, fused at base, yellow, ca. 4 mm long. **Capsules** nodding, ca. 10 mm long, nearly glabrous, unequally 3-winged, abaxial wing crescent-shaped, ca. 4 mm high.

### Distribution and ecology

Known only from the type locality in Guangxi, China. On limestone hill.

### Phenology

Flowering from March to May; fruiting in May.

### Etymology

This species is named in honor of Mr. Shi-Nian Lu (陆仕念), an engineer of Jingxi Forestry Bureau of Guangxi and an expert of Jingxi’s flora who has helped tremendously the exploration of Guangxi’s limestone flora.

### Additional specimen examined (paratypes)

CHINA. Guangxi, Jingxi County, Biaolin Town, Longjing, on rocks of limestone hill, 25 Aug 1935, in flowers and with dry fruit, *S. P. Ko 55623* (IBSC!, paratype of *Begonia bonii* var. *remotisetulosa*); Baise City, Jingxi County, Wuping Town, Yixing Village, Pai Lin Tun, Baiyan Cave, elev. 730 m, 23°09’28”N, 106°35’4”E, on limestone hill, 22 May 2007, *Ching*-*I Peng 21111*, with *Yan Liu*, *Hai*-*Shan Gao*, *Kuo*-*Fang Chung*, *Ming*-*Chao Yu*, and *Shi*-*Nian Lu* (HAST-117599), *Ching*-*I Peng* et al*. 21112* (HAST-117600).

### Notes

*Begonia lui* was first proposed in S.-M. Ku’s master thesis (Ku 2006) as *Begonia koi* S.M.Ku et al., sp. nov. ined. based on a single collection *S. P. Ko 55623* (IBSC). This specimen was cited in Shui and Chen (2005) as the paratype of *Begonia bonii* var. *remotisetulosa* Y.M.Shui & W.H.Chen, which was treated as a synonym *Begonia debaoensis* C.I Peng, Yan Liu & S.M.Ku (Ku et al. 2006) in the Flora of China (Gu et al. 2007). Because *S. P. Ko 55623* differs considerably from the holotype of *B. bonii* var. *remotisetulosa* [*Y. M. Shui et W. H. Chen B2004*-*91* (KUN!)] by its thicker rhizomes, longer internodes, larger leaf blades, white maculation in intercostal areas, larger fruits, and rectangular capsule wing, Ku (2006) named the collection as *B. koi* after its collector *S. P. Ko* (高錫朋). However, despite our continuous effort in searching for the plant, the collecting locality of *S. P. Ko 55623* [廣西省 (Guangxi Province) 靖西縣 (Jingxi County) 表林鄉 (Biaolin Town) 隴徑 (Longjing)] could not be located and the species remained insufficiently known. This puzzle was resolved when the population in Wuping Town was discovered by Mr. Shi-Nian Lu.

Morphologically, *Begonia lui* somewhat resembles *B. crystallina* Y.M.Shui & W.H.Chen from Yunnan in the leaf shape and indumentum (Shui and Chen 2005); however, the leaf size of the former species is smaller than the latter. Additionally, *B. lui* has slightly rugose leaves, persistent stipules, shorter petioles and larger flowers that can be distinguished from *B. crystallina*.

**Fig. 12:**
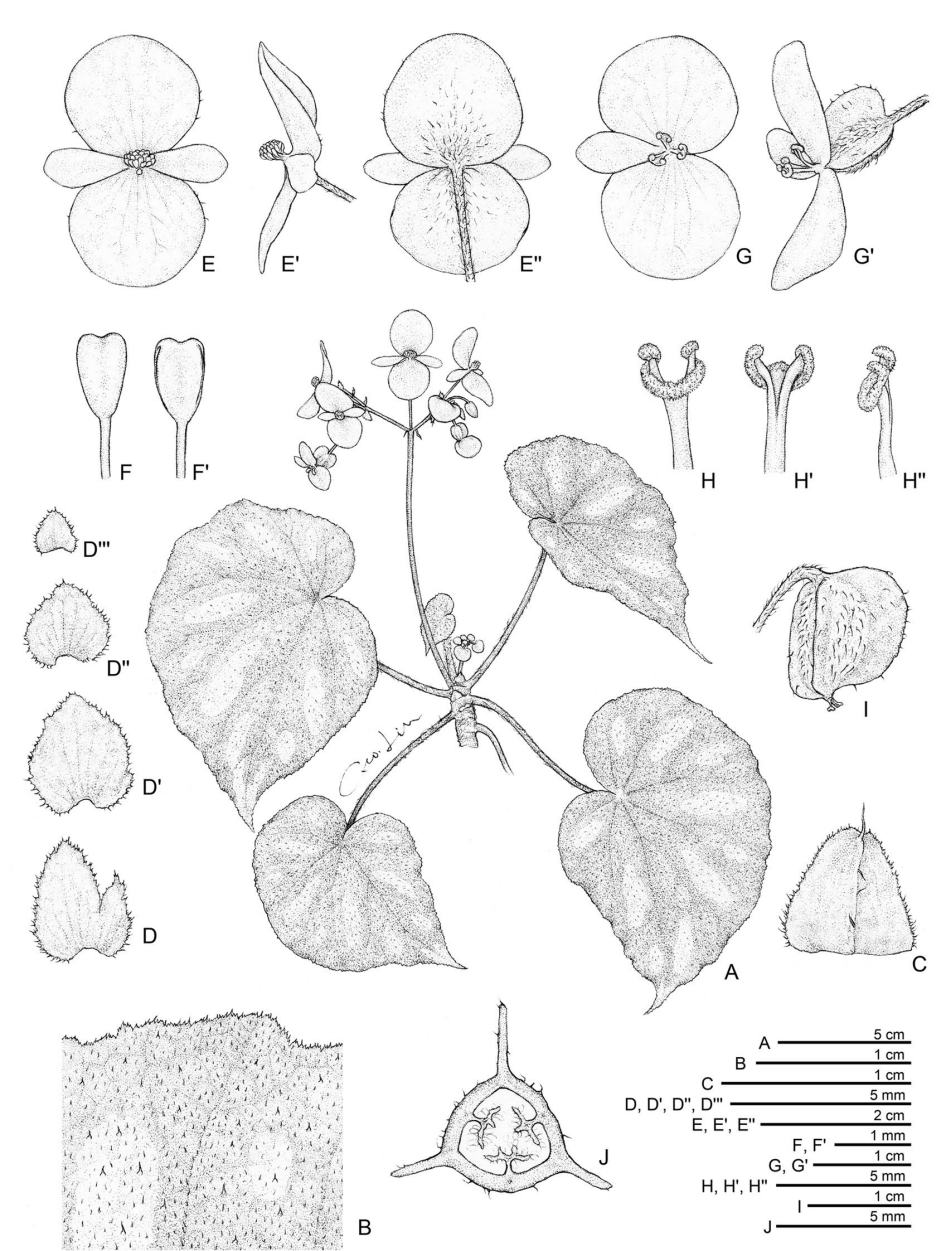
*Begonia scabrifolia* C.I Peng, Yan Liu & C.W.Lin. **A** Habit; **B** Portion of adaxially leaf; **C** Stipule; **D**, **D’**, **D’’**, **D’’’** Bracts, lower to upper; **E**, **E’**, **E’’** Staminate flower, face, side and back views; **F**, **F’** Stamen, dorsal and ventral views; **G**, **G’** Pistillate flower, face and side views; **H**, **H’ H’’** Style and stigmatic band, dorsal, ventral and side views; **I** Capsule; **j** Cross section of an immature capsule. All from *Peng 22197*

**Fig. 13:**
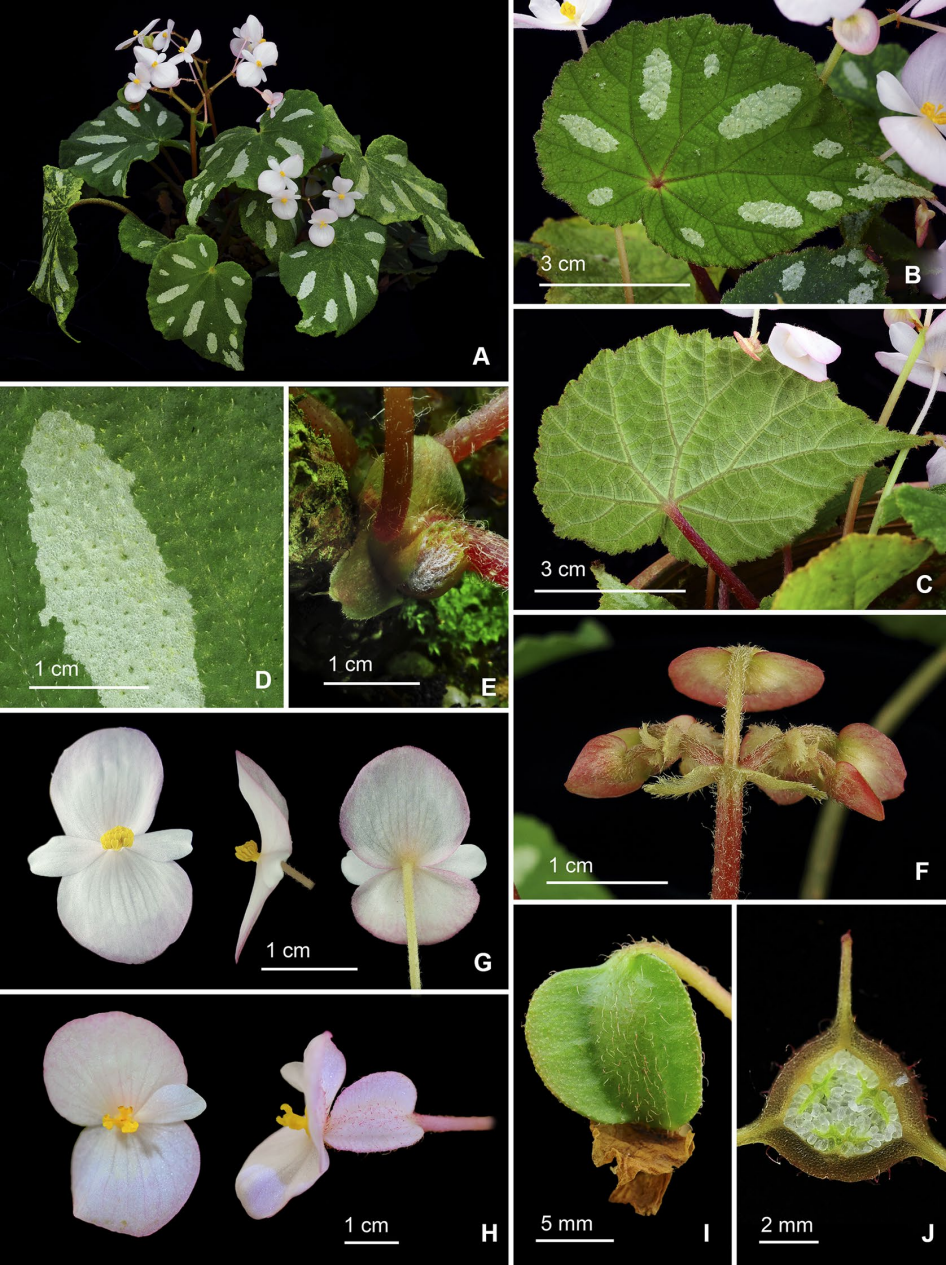
*Begonia scabrifolia* C.I Peng, Yan Liu & C.W.Lin. **A** Cultivated plant at anthesis; **B** Leaf adaxial surface; **C** Leaf abaxial surface; **D** Leaf adaxial surface, showing indumentum; **E** Stipules; **F** Early stage of inflorescence, showing bracts; **G** Staminate flowers; **H** Pistillate flowers; **I** Fruit; **J** Cross section of ovary. All from *Peng 22197*

**5.*Begonia scabrifolia*** C.I Peng, Yan Liu & C.W.Lin, sp. nov. (Sect. *Coelocentrum*) 澀葉秋海棠 (Figs. 12 and 13).

**Type:** CHINA. Guangxi, detailed locality unknown, cultivated in Guilin Botanical Garden, plant collected on 18 May 2009, type specimens (in flowers) pressed from plants cultivated in the experimental greenhouse, Academia Sinica, Taiwan, *Ching*-*I Peng 22197* (holotype: IBK; isotype: HAST-144969).

Monoecious rhizomatous herb. **Rhizomes** stout, creeping, to 10 cm or longer, 7–15 mm thick, internodes congested, subglabrous. **Stipules** persistent, pale yellowish green to reddish brown, triangular-ovate, 6–11 mm long, 6–9 mm wide, herbaceous, strongly keeled, margin fimbricate, apex aristate, arista 1–3 mm long. **Leaves** alternate; petiole terete, yellowish green to crimson, 4.5 − 15 cm long, 3–4 mm thick, white villous or sericeous; leaf blade asymmetric, oblique, widely ovate, 9 − 14.5 cm long, 6–8.5 cm wide, broad side 4–6 cm wide, basal lobes cordate, 3.7–4.5 cm long, apex acuminate to shortly caudate, margin denticulate and densely scabrous; leaf thick chartaceous, adaxially bright green, dark green to brownish green, sometimes embellished with crushing silvery white striped between primary and secondary veins; surface shortly scabrous, hair white; abaxially pale green, scabrous on all veins; venation basally ca. 7 palmate, midrib distinct, ca. 3 secondary veins on each side, tertiary veins percurrent or reticulate. **Inflorescences** axillary, dichasial cymes, arising directly from rhizome, branched 2 or 3 times; peduncle pale green to red, 5–10 cm long, velutinous; bracts persistent, pale green, ovate to widely ovate, sometimes with 1 or 2 lobes, first pair 5–7 mm long, 2.5–6 mm wide, margin fimbriate, bracts of upper inflorescence similar but smaller. **Staminate flower:** pedicel 1.5–2.5 cm long, sericeous, tepals 4, white to pinkish; outer 2 very widely ovate to suborbicular, 12–18 mm long, 16–20 mm wide, abaxially sericeous, inner 2 obovate 10–13 mm long, 4–6 mm wide; androecium zygomorphic, 4–5 mm across; stamens golden yellow, 17–33; filaments shortly fused at base; anthers obovate, ca. 1.2 mm long, 2-locular, apex retuse, subequal at filaments. **Pistillate flower:** pedicel 1.5–2 cm long, sericeous, tepals 3, white to pinkish, outer 2 suborbicular, 11–15 mm long, 12–18 mm wide, abaxially sericeous; inner 1 narrowly oblong to elliptic, 8–10 mm long, 4–5 mm wide, glabrous; ovary widely ellipsoid, 5–7 mm long, 2.5–4 mm thick (wings excluded), pinkish, sericeous; 3-winged, wings unequal, yellowish green to pinkish, narrowly crescent-shaped, lateral wings 2, narrowly, ca. 2 mm high, abaxial wing 4 mm high, margin entire, sericeous; styles 3, fused at base, yellow, ca. 5 mm long, stigma spirally twisted. **Capsule:** tepals persistent; capsule body ellipsoid, ca. 1 cm long, 5 mm thick (wings excluded), greenish when fresh; wings unequal, crescent-shaped, lateral wings 2, ca. 3.5 mm high, abaxial wing 4.5 mm high.

### Distribution and ecology

*Begonia scabrifolia* has long been cultivated in Guilin Botanical Garden, Guangxi. Its precise origin is not known.

### Etymology

The species epithet refers to the rough and scabrous leaf surface of the adaxial side.

### Notes

*Begonia scabrifolia* is similar to *B. bamaensis* Yan Liu & C.I Peng (Liu et al. 2007) in their variegated ovate leaves, white to pinkish tepals and zygomorphic androecium, differing in its persistent stipules and bracts (vs. caducous), sericeous pedicels, abaxial sides of outer tepals (vs. pilose), and straight abaxial wing of capsules (vs. markedly curved toward one side). Phylogenetically, *B. scabrifolia* is sister to *B. lui* and together they form the sister clade of *B. bamaensis* (Fig. 2). As *B. scabrifolia* is only known from a cultivated plant in Guilin Botanical Garden without collecting locality, we are hoping that the description of this species can stimulate exploration to areas adjacent to the type localities of *B. lui* and *B. bamaensis* for the wild population of *B. scabrifolia*.

**Fig. 14:**
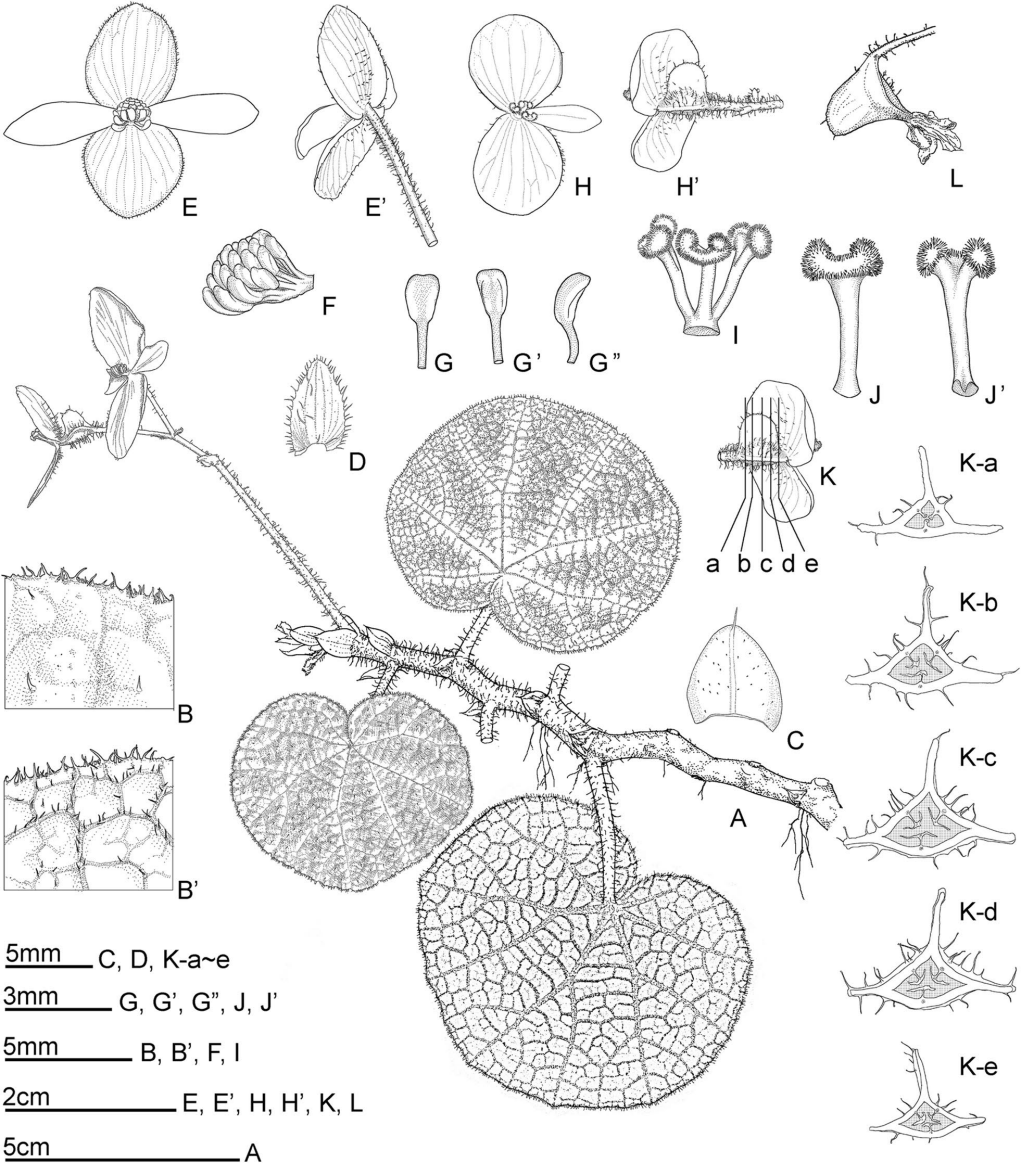
*Begonia zhuoyuniae* C.I Peng, Yan Liu & K.F.Chung. **A** Habit; **B**, **B’** Portion of leaf, showing indumentum on adaxial and abaxial surfaces; **C** Stipule; **D** Bract; **E**, **E’** Staminate flower, face and back views; **F** Androecium, side view; **G**, **G’**, **G”** Stamens; **H**, **H’** Pistillate flower, face and back views; **I**, **J**, **J’** Style and Stigmas; **K-A**–**K-E** Serial cross sections of ovary; **L** Fruit. All from *Peng* et al*. 20737*-*A*

**Fig. 15:**
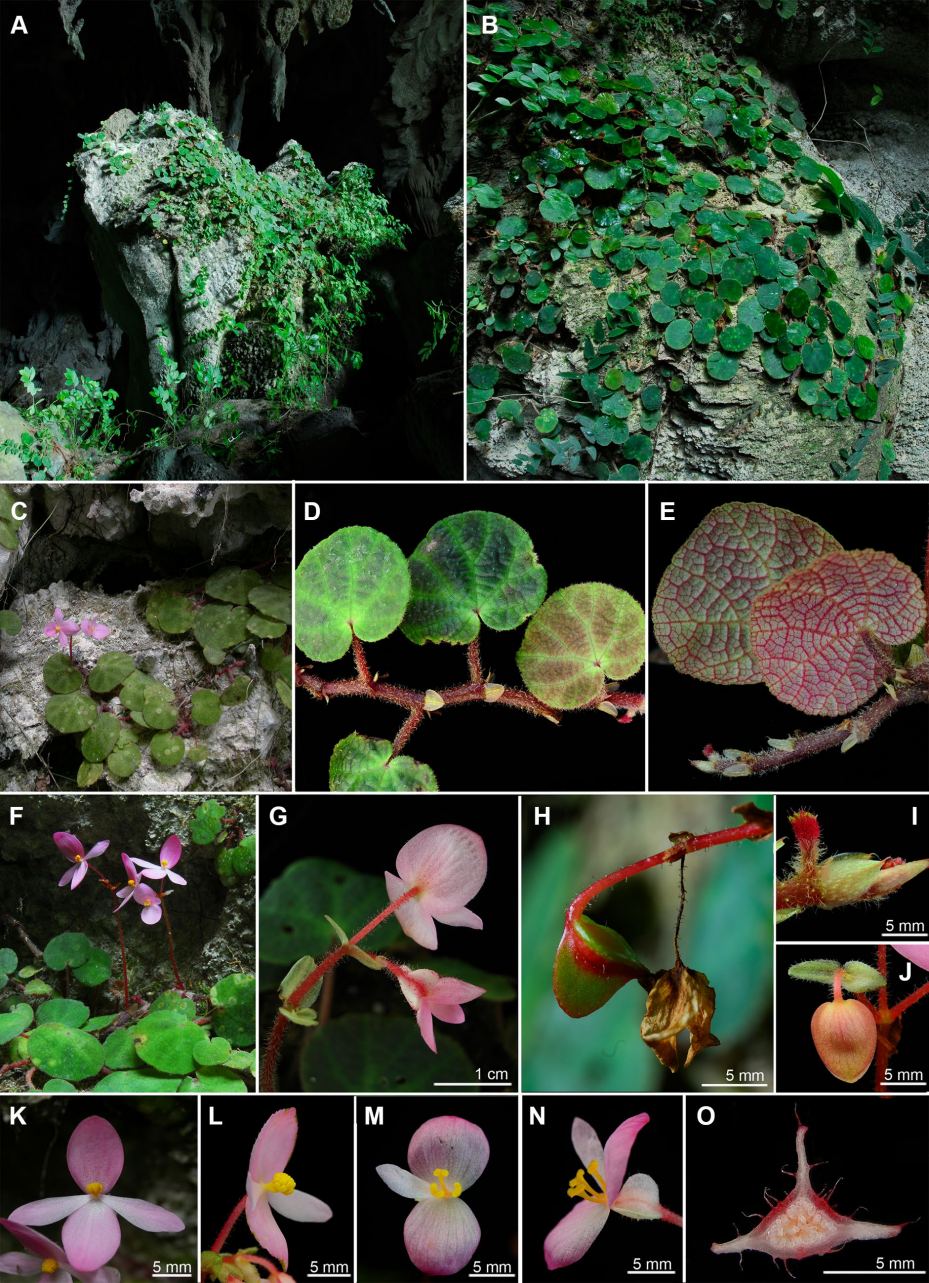
*Begonia zhuoyuniae* C.I Peng, Yan Liu & K.F.Chung. **A**–**C**, **F** Habit and habitat; **D** Rhizome and leaf adaxial surfaces; **E** Leaf abaxial surfaces; **G** Inflorescence, showing indumentum on tepals and pedicels; **H** Developing fruit; **I** Stipule; **J** Staminate flower bud and bracteoles; **K**, **L** Staminate flower; **M**, **N** Pistillate flower; **O** Cross section of ovary. **A**, **B**, **H** from C.-I *Peng 21061*; **C**, **F**, **K** field photos taken by Yan Liu without voucher; **D**, **E**, **I**, **J**, **L**–**O** from *C.*-*I Peng 20737*-*A* (HAST); **G** from *S.*-*M. Ku 2024*

**6.*Begonia zhuoyuniae*** C.I Peng, Yan Liu & K.F.Chung, sp. nov. (Sect. *Coelocentrum*) 倬雲秋海棠 (Figs. 14 and 15).

**Type:** CHINA, Guangxi, Donglan County, ca. 6.6 km S of Wuzhuan, 3.5 km S of Baxue Tun, on rocky slope in shaded limestone cave, elev. ca. 400 m, 24°57’54”N, 107°18’53”E, plant collected on 18 December 2005, type specimens (in flowers) pressed from plants cultivated in the greenhouse, Academia Sinica, Taiwan, *Ching*-*I Peng 20737*-*A* with *Yan Liu*, *Shin*-*Ming Ku*, *Tsung*-*Han Tsai* (holotype: IBK; isotype: E, HAST-144991, K, KUN, PE).

Monoecious rhizomatous herb. **Rhizomes** creeping, slender, to 80 cm long, 2–6 mm across, internodes 5–25 mm long, purple red, pilose. **Stipules** persistent, ovate, ca. 5 mm long, 5 mm wide, abaxially sparsely red glandular-puberulent, slightly keeled, apex aristate, arista 1 mm long, margin entire. **Leaves** alternate; petiole terete, 1.5–4.5 cm long, 2 mm across; leaf blade asymmetric, ovate to reniform, 2.5–5(–6.5) cm long, 2–4(–5.5) cm wide, apex rounded or obtuse, base cordate, chartaceous, adaxially somewhat rugose, pale green or dark green with purple red between veins, sparsely hirtellous and punctate, abaxially red on veins and somewhat reddish, densely pilose to tomentose, margin crenulate and ciliate. **Inflorescences** axillary, arising directly from rhizome, cymes monochasial or dichasial, branched 1–3 times. Peduncle 4.5–10.5 cm long, 1.5 mm across, pilose; bracts persistent, elliptic, 4–6 mm long, 2 mm wide, light green, nearly glabrous, margin ciliate. **Staminate flower**: pedicel 1.2–1.7 cm, glandular-pilose, tepals 4, outer 2 ovate, suborbicular or elliptic, ca. 1.2–2 cm long, 1–1.4 cm wide, pink, abaxially pilose, margin sparsely ciliate, inner 2 elliptic or oblanceolate, 1.3–1.5 cm long, ca. 4 mm wide, pinkish white; androecium zygomorphic, ca. 4 mm across, stamens 16–28, anthers oblong-obvoid, 2-locular, connective apex emarginate. **Pistillate flower**: pedicel ca. 1.1 cm long, glandular-pilose, tepals 3, outer 2 ovate to suborbicular, 9 mm long, 8 mm wide, pink, margin sparsely ciliate, inner 1 obovate, 7 mm long, 3 mm wide, pinkish white, ovary trigonous-ellipsoid, 5–10 mm long, ca. 3 mm thick (wings excluded), sparsely glandular-pilose, 3-winged; wings sub-equal, abaxial wing 3 mm high, lateral wings ca. 2 mm high, pinkish white; styles 3, 1.5–4 mm long, free, stigma apically C-shaped. **Capsules** trigonous-ellipsoid, ca. 8 mm long, ca. 4 mm thick (wings excluded); abaxial wing crescent-shaped, ca. 4 mm high.

### Chromosome cytology

Somatic chromosomes of *Begonia zhuoyuniae* at metaphase were counted as 2*n* = 30 (Fig. 9b), identical to the number reported by Han et al. (2018) as well as majority of species of Sect. *Coelocentrum* (Chung et al. 2014). The length of chromosomes varied from ca. 0.8 to 1.3 µm. The centromere positions of several small chromosomes could not be determined. However, most chromosomes were metacentric. Satellites were not observed.

### Distribution and ecology

Known only from two limestone caves in Donglan County and Bama Yao Autonomous County, northwestern Guangxi.

### Phenology

Flowering from February to May; fruiting from April to July.

### Etymology

The specific epithet honors the visionary Dr. Cecilia Koo Yan Zhuo-yun (辜嚴倬雲), founder of Dr. Cecilia Koo Botanic Conservation Center (KBCC) that aims to conserve tropical and subtropical plants and maintain earth’s rich biodiversity.

### Additional specimens examined (paratypes)

CHINA. Guangxi, Bama Yao Autonomous County, Xishan Town, Bana Village, Nongna Tun, elev. 500–550 m, 24°14’17”N, 107°15’50”E, 23 December 2005, *Shin*-*Ming Ku 2024* (HAST-144990); Donglan County, Wuzhuan Town, at 18 km road maker on Xian Rd. 895, elev. 430 m, 24°17’54”N, 107°18’15”E, limestone mountain, E-facing cave, 16 May 2007, *Ching*-*I Peng 21061* with *Yan Liu*, *Hai*-*Shan Gao*, *Kuo*-*Fang Chung* & *Ming*-*Chao Yu* (HAST-117106).

### Notes

*Begonia zhuoyuniae* somewhat resembles *B. aurantiflora* C.I Pen, Yan Liu & S.M.Ku (Peng et al. 2008) in the elongated rhizomes, differing by its smaller leaves (2.5–5 × 2–4 vs. 7–11 × 5.5–10 cm) and pink tepals (vs. orange). *Begonia zhuoyuniae* is also similar to *B. semiparietalis* Yan Liu, S.M.Ku & C.I Peng (Ku et al. 2006) but can be distinguished from the latter by the thinner (2–6 vs. 9–12 mm) and generally elongated rhizomes (5–25 vs. 4–12 mm), smaller leaves (2.5–5 × 2–4 vs. 8–12 × 6–10 cm), scabrous (vs. pilose) abaxial leaf surface, and hairy (vs. glabrous) peduncles. A detailed comparison of the three species is provided in Table 3.

**Table 3:** Comparison of *Begonia zhuoyuniae* with *B. aurantiflora* and *B. semiparietalis*

	*Begonia zhuoyuniae*	*B. aurantiflora* (Peng et al. 2008)	*B. semiparietalis* (Ku et al. 2006)
Rhizome			
Diameter (mm)	2–6	3–6	9–12
Internode length (mm)	Elongated, 5–25	Elongated, 12–50	Congested, 4–17
Leaf			
Shape	Ovate to reniform, apex rounded or obtuse, base cordate	Broadly ovate or suborbicular, apex obtuse to acute, base deeply cordate	Broadly ovate or suborbicular, apex shortly acuminate or acute, rarely obtuse, base deeply cordate
Size (cm)	2.5–5(–6.5) × 2–4(–5.5)	(4.5–)7–11 × (4–)5.5–10	(3.5–)8–12(–15) × (3–)6–10(–13)
Adaxial surface	Sparsely hirtellous and punctate	Pilose	Pilose-setose (trichomes 0.7–1 mm long)
Abaxial surface	Scabrous	Pilose	Pilose
Inflorescence			
Peduncle	Pilose	Glandular-pilose	Glabrous
Tepal color	Pink	Orange	Pinkish or reddish

## Supplementary information

**Additional file 1:** Taxon sampling and NCBI accession numbers.

**Additional file 2:** DNA sequence alignment.

## Data Availability

NCBI accessions numbers of DNA sequences downloaded from GenBank are summarized in Additional file 1. DNA sequence alignment is available as Additional file 2.
