# Supplementary material for: Six new species of Begonia from Guangxi, China
Source: Bot Stud. 2020 Jul 30;61:21. doi: 10.1186/s40529-020-00298-y (PMC7393003; doi:10.1186/s40529-020-00298-y)
Supplement: Supplementary file 1 — Additional file 1: Taxon sampling and NCBI accession numbers. [file 40529_2020_298_MOESM1_ESM.docx]

**Additional file 1**: Taxon sampling and NCBI accession numbers. Sect. *Name* (§*Abbreviation*)—Species: NCBI accession numbers (*ndhA*/*ndhF-rpl32*/*rpl32-trnL*).

*Begonia* sect. *Alicida* C.B.Clarke (§*ALI*)—*B. alicida* C.B.Clarke: JF756388/JF756472/JF756556; Sect. *Baryandra* A.DC. (§*BAR*)—*B. blancii* M.Hughes & C.I Peng: KR186450/KR186537/KR186711, *B. fenicis* Merr.: KR186464/KR186551/KR186724, *B. hughesii* Rubite & C.I Peng: KR186477/KR186564/KR186737, *B. longiscapa* Warb.: KR186482/KR186569/KR186742, *B. luzonensis* Warb.: KR186484/KR186571/KR186744, *B. rufipila* Merr.: KR186506/KR186593/KR186766; Sect. *Coelocentrum* Irmsch. (§*COE*)—*B. leprosa* Hance: –/–/MH235433, *B. masoniana* Irmsch.: JF756372/JF756456/JF756540, *B. morsei* Irmsch.: JF756373/JF756457/JF756541, *B. ningmingensis* D.Fang, Y.G.Wei & C.I Peng: KR186500/KR186587/KR186760, *B. pseudodryadis* C.Y.Wu: MH207317/MH207731/–, *B. pulvinifera* C.I Peng & S.M. Ku: MH207322/MH207735/–, *B. scabrifolia* C.I Peng, Yan Liu & C.W.Lin: MT571496/MT571497/MT571498, *B. sinofloribunda* Dorr: MH207363/MH207778/MH208176, *B. variegata* Y.M.Shui & W.H. Chen: –/–/MH208225; Sect. *Diploclinium* (Lindl.) A.DC. (§*DIP*): *B. aceroides* Irmsch.: JF756385/JF756469/JF756553, *B. brandisiana* Kurz: JF756379/JF756463/JF756547, *B. demissa* Craib: JF756384/JF756468/JF756552, *B. flagellaris* H.Hara: MH207147/MH207558/MH207972, *B. gigabracteata* H.Z.Li & H.Ma: MH207165/MH207577/MH207991, *B. grandis* Dryand: JF756351/JF756435/JF756519, *B. lithophila* C.Y.Wu: MH207225/MH207635/MH208049, *B. murina* Craib: MH207255/MH207667/MH208077, *B. picta* Sm.: MH207294/MH207708/MH208112, *B. poilanei* Kiew: MH207305/MH207719/MH208122, *B. puttii* Craib: KP712959/KP713201/KP713326, *B. rabilii* Craib: KP712968/KP713164/KP713328, *B. rubella* Buch.-Ham. ex D.Don: MH207336/MH207749/MH208148, *B. tribenensis* C.R.Rao: MH207404/MH207826/MH208217, *B. wilsonii* Gagnep.: MH207423/MH207844/MH208231, *B. yunnanensis* H.Lév.: MH207426/MH207847/MH208234; Sect. *Haagea* (Klotzsch) A.DC. (§*HAA*)—*B. dipetala* Graham.: JF756341/JF756425/JF756509; Sect. *Lauchea* (Klotzsch) A.DC. (§*LAU*)—*B. pteridiformis* Phutthai: MH207319/MH207732/MH208132; Sect. *Parvibegonia* A.DC. (§*PAR*)—*B. sibthorpoides* Ridl.: MH207359/MH207774/MH208171, *B. tenuifolia* Dryand: JF756349/JF756433/JF756517; Sect. *Petermannia* (Klotzsch) A.DC. (§*PET*)—*B. amphioxus* Sands: MH207038/MH207449/MH207865, *B. baik* C.W.Lin & C.I Peng: MH207050/–/MH207875, *B. burbidgei* Stapf: MH207080/MH207490/MH207907, *B. doloisii* Rimi: MH207134/–/MH207957, *B. imbricata* Sands: MH207197/MH207607/MH208022, *B. rubida* Ridl.: MH207337/MH207750/MH208149, *B. vaccinioides* Sands: MH207413/MH207836/MH208223; Sect. *Platycentrum* (§*PLA*)—*B. abdullahpieei* Kiew: MH207018/MH207427/MH207848, *B. aborensis* Dunn: MH207019/MH207428/MH207849, *B. acetosella* Craib: JF756367/JF756451/JF756535, *B. aptera* Blume: JF756369/JF756453/JF756537, *B. areolata* Miq: MH207043/MH207453/MH207869, *B. balansana* Gagnep.: MH207051/MH207460/MH207876, *B. baviensis* Gagnep.: MH207054/MH207464/MH207880, *B. cathcartii* Hook.f.: MH207089/MH207499/MH207915, *B. ceratocarpa* S.H.Huang & Y.M.Shui: MH207090/MH207500/MH207916, *B. decora* Stapf: JF756355/JF756439/JF756523, *B. diadema* Linden ex Rodigas: MH207129/MH207539/MH207952, *B. dux* C.B.Clarke: MH207137/MH207546/MH207960, *B. griffithiana* (A.DC.) Warb.: MH207172/MH207582/MH207998, *B. handelii* Irmsch.: MH207176/MH207586/MH208002, *B. hatacoa* Buch.-Ham. ex D.Don: JF756354/JF756438/JF756522, *B. koksunii* Kiew: MH207214/MH207624/MH208038, *B. limprichtii* Irmsch.: MH207223/MH207633/MH208047, *B. longifolia* Blume: JF756368/JF756452/JF756536, *B. multangula* Blume: JF756364/JF756448/JF756532, *B. nepalensis* (A.DC.) Warb.: MH207257/MH207669/MH208079, *B. obovoidea* Craib: JF756386/JF756470/JF756554, *B. palmata* D.Don: JF756360/JF756444/JF756528, *B. pavonina* Ridl.: JF756356/JF756440/JF756524, *B. pedatifida* H.Lév.: MH207288/MH207700/MH208105, *B. perakensis* King: MH207291/MH207704/MH208108, *B. rhoephila* Ridl., MH207331/MH207745/MH208143, *B. robusta* Blume: JF756363/JF756447/JF756531, *B. roxburghii* (Miq.) A.DC.: JF756371/JF756455/JF756539, *B. scottii* Tebbitt: MH207347/MH207761/MH208159, *B. sikkimensis* A.DC.: JF756359/JF756443/JF756527, *B. silletensis* (A.DC.) C.B.Clarke: JF756370/JF756454/JF756538, *B. sizemoreae* Kiew: JF756361/JF756445/JF756529, *B. thomsonii* A.DC.: MH207398/MH207818/MH208210, *B. venusta* King: JF756357/JF756441/JF756525, *B. versicolor* Irmsch.: JF756358/JF756442/JF756526; Sect. *Pritzelia* Klotzsch A.DC. (§*PRI*)—*B. rigida* Linden ex Regel: MH207332/MH207746/MH208144; Sect. *Reichenheimia* (Klotzsch) A.DC. (§*REI*)—*B. floccifera* Bedd.: JF756343/JF756427/JF756511, *B. hymenophylla* Gagnep.: JF756382/JF756466/JF756550; Sect. *Tatraphila* A.DC. (§*TAT*)—*B. komoensis* Irmsch.: MH207215/MH207625/MH208039; Species unassigned to section (§*Ignota*)—*B. boisiana* Gagnep.: MH207060/MH207471/MH207888.
